# From Classification to Clinical Trust: 3D Deep Learning and Layer-Wise Explainability for Brain Tumor MRI

**DOI:** 10.3390/s26216993

**Published:** 2026-11-05

**Authors:** Nikolaos Pagonis, Konstantinos Plomaritis, Evangelos Kogias, Nikolaos Foroglou, Ioanna Chouvarda

**Affiliations:** 1 Laboratory of Computing, Medical Informatics and Biomedical Imaging Technologies, School of Medicine, Aristotle University of Thessaloniki, 541 24 Thessaloniki, Greece; kplomarb@auth.gr (K.P.); ioannach@auth.gr (I.C.); 2 Department of Neurosurgery, AHEPA University Hospital, School of Medicine, Aristotle University of Thessaloniki, 541 24 Thessaloniki, Greece; kogias@auth.gr (E.K.); nforoglou@auth.gr (N.F.)

**Keywords:** explainable AI, brain tumor classification, 3D convolutional neural network, Grad-CAM, glioma, meningioma, attention mechanism, medical-image interpretability

## Abstract

**Highlights:**

**What are the main findings?**

• A 3D multimodal deep learning pipeline (ResNet-34 with CBAM attention) achieved strong, well-calibrated glioma–meningioma classification performance (96% accuracy, 0.993 AUC, ECE = 0.0165) from four-modality MRI.
• A novel reconstruction-decoder approach yields full-resolution, decoder-mediated attribution that integrates the network’s lowest-resolution bottleneck, where standard Grad-CAM is too coarse to be informative, with higher-resolution skip features, without requiring segmentation supervision.
• Four quantitative metrics (faithfulness, spatial localization, progressive masking, and inter-method agreement) provide complementary views of attribution quality. The decoder-assisted Layer 4 is strongest on localization and deletion, whereas Layer 1 is higher on insertion and Layer 2 on overall faithfulness.

**What are the implications of the main findings?**

• The proposed decoder is a general, annotation-free strategy applicable to any 3D CNN facing deep-layer resolution collapse, offering a practical path toward more trustworthy explainability in volumetric medical imaging beyond this specific classification task.
• These results motivate routine per-class, per-layer validation of attribution methods in future medical imaging XAI work, rather than relying on single, aggregate faithfulness scores.

**Abstract:**

Preoperative differentiation of glioma from meningioma on MRI is important for treatment planning but depends on expert interpretation. A 3D ResNet-34 with CBAM attention was trained on four-modality MRI from 2251 BraTS 2023 patients (1801 for four-fold cross-validation; 450 held out for testing) and achieved 96% accuracy, an AUC of 0.993, and an expected calibration error of 0.0165 on the test set. For layer-wise explanation, Grad-CAM and SmoothGrad were applied at the four residual stages, and a reconstruction decoder was added to obtain full-resolution, decoder-mediated attribution at the 4 × 4 × 4 bottleneck, where standard Grad-CAM is too coarse. Four quantitative metrics gave complementary results: the decoder-assisted attribution had the highest pointing-game accuracy (80.2%) and the lowest deletion AUC, whereas Layer 1 had the highest insertion AUC and Layer 2 the highest mean faithfulness delta, with no significant faithfulness difference among Layers 1, 2, and 4. Glioma was localized by most layers, while meningioma was localized mainly through the decoder-assisted pathway. A preliminary clinical review and a normalization analysis are also reported. The decoder offers an annotation-free way to obtain full-resolution attribution from deep layers of volumetric models.

## 1. Introduction

Among the most frequent and deadly cancers in children, brain tumors are also highly prevalent among adults [1,2]. In terms of origin, and clinical and radiologic presentation, the two most prevalent tumor types, namely glioma and meningioma, are essentially different, as the former is always infiltrative and malignant and needs urgent treatment [3,4], while the latter usually forms a distinct mass and is commonly managed non-aggressively [3,5]. Nevertheless, in both gliomas and meningiomas, there are some overlapping signs and imaging features, such as identical contrast enhancement and peritumoral edema, which introduce challenges in their differential diagnosis [6,7,8]. This inconsistency is highly problematic for clinical practice, as the treatment of glioma for meningioma or vice versa can be dangerous for patients [9,10,11,12,13].

Deep learning, more specifically convolutional neural networks (CNNs), has been successful in the field of medical imaging classification, especially brain tumor classification through MRI images [11,12,13]. For instance, a customized CNN combined with an optimized ResNet101 architecture has achieved high classification accuracy across four brain tumor categories (glioma, meningioma, pituitary, and no tumor) from MRI data [14]. Similarly, lightweight CNN architectures have demonstrated strong diagnostic performance even under limited training-data availability, a common constraint in medical imaging datasets [15]. Several additional 2D CNN studies report comparably strong performance on glioma–meningioma classification tasks, frequently exceeding 96–99% accuracy [16,17], confirming that raw classification performance in this task is already high in the existing literature.

Volumetric 3D CNN approaches for brain tumor classification remain notably rare in the literature, despite the clear advantages of preserving spatial and structural information across the full MRI volume. Where 3D approaches do exist, they are largely confined to single-tumor-type tasks and, in several cases, a single MRI modality: glioma grading using multi-scale 3D CNNs trained exclusively on T1 contrast-enhanced images [18], glioma grading combined with healthy-brain discrimination using 3D ResNet-based “spatiospatial” architectures, likewise restricted to a single MR contrast [19], and molecular subtyping (IDH/MGMT status) within glioma specifically, using a 3D CNN ensemble with attribution- based explainability via Integrated Gradients and Shapley values [20]. This reliance on a single modality forgoes the complementary diagnostic information carried by different MRI sequences (Section 2.2), a limitation the present study addresses directly through four-modality multimodal fusion. Despite an extensive search of the literature, remarkably few 3D CNN classification studies for brain tumors were identified overall, underscoring how underexplored volumetric classification approaches remain for this domain relative to their well-established use in the closely related task of segmentation.

Explainability is increasingly incorporated into brain tumor classification studies, most commonly via Grad-CAM applied to 2D CNN pipelines, frequently paired with a second method, such as SHAP, to provide complementary region- and feature-level interpretability [21]. Some studies extend beyond simple visual inspection: Gharaibeh (2025) additionally conducted a consistency analysis checking attribution maps against expected anatomical tumor locations, and a structured radiologist review rating explanation relevance on a Likert scale [21]. While methodologically more rigorous than purely qualitative presentation, such validation remains fundamentally expert-agreement-based, assessing whether an explanation looks plausible to a human rater, rather than model behavior-based, assessing whether the model’s actual output changes when the highlighted region is perturbed. To the best of our knowledge, no prior study, 2D or 3D, applies faithfulness, spatial localization, progressive masking, or inter-method quantitative agreement metrics to validate brain tumor classification explanations, the specific form of validation rigor that this work introduces.

The most closely related work identified is a 3D CNN classifying seven intracranial tumor classes, including both glioma (as high- and low-grade subtypes) and meningioma, alongside brain metastasis, pituitary adenoma, acoustic neuroma, and healthy tissue [22]. This study demonstrates that 3D volumetric classification across multiple tumor types, including meningioma, is both feasible and effective (internal test accuracy, 93.35%; meningioma AUC, 1.00). However, it addresses meningioma as one of seven classes within a broader multi-tumor classification problem, rather than as part of a dedicated, systematically validated glioma–meningioma discrimination task, and its explainability analysis, while using Grad-CAM, remains qualitative, described by the authors themselves as producing “coarse attention maps,” with no faithfulness, spatial localization, or inter-method agreement validation applied. No study was identified that applied a 3D CNN specifically to binary glioma–meningioma discrimination with systematically validated explainability, the specific intersection that this study occupies. Table 1 summarizes these dimensions of comparison alongside the present study.

Yet, classification accuracy alone is not sufficient to support clinical use, because without the capability of explaining its rationale, there is no way that a clinician can trust, question, and apply its prediction, an issue that is being raised by regulations on interpreting the results of diagnostics. Grad-CAM, which belongs to saliency mapping-based explainable methods, has been the golden standard for this purpose [23,24,25].

A significant limitation of these methods, however, remains largely unaddressed in the literature: standard Grad-CAM-style attribution degrades substantially at the deepest layers of a convolutional network, precisely where the most class-discriminative, semantically rich features reside. This degradation arises because spatial resolution collapses through successive downsampling stages, leaving deep-layer feature maps too spatially coarse to be meaningfully resampled back to input resolution, an issue compounded in three-dimensional architectures operating on volumetric MRI, where existing explainability work remains comparatively rare and almost entirely unvalidated beyond qualitative visual inspection [23,24,25,26,27].

The loss of spatial resolution in deep features is not specific to medical imaging, and it has been addressed in several fields. In natural-image classification, gradient-based class activation methods are known to yield coarse maps at deep layers. Refinements either combine gradients from shallower, higher resolution layers or examine how large receptive fields limit localization [28]. In remote sensing, where images contain rich spatial detail and objects at many scales, coarse high-level maps have motivated explanation methods that recover detailed spatial information [29]. Similar localization concerns have been studied for defect classification in semiconductor manufacturing [30]. In dense prediction, Grad-CAM has been extended to semantic segmentation [31] and learned feature upsampling has been proposed to restore the resolution that pooling removes from deep vision features while preserving their semantics [32]. In video and volumetric networks, explaining 3D-CNN predictions raises additional difficulties, such as following moving structures across frames [33]. These approaches rely on shallower layers, on additional learned upsampling, or on task-specific modifications [34].

To address this gap, this study applies a systematically validated, multi-method explainability pipeline to a 3D deep learning model for glioma–meningioma classification from multimodal MRI and introduces a novel reconstruction-decoder approach to resolve resolution collapse at the network’s deepest layer without requiring additional segmentation supervision. The contribution of this work is fourfold: (1) a 3D multimodal deep learning classification pipeline for glioma–meningioma discrimination from four-modality MRI, incorporating CBAM attention modules, achieving strong and well-calibrated performance (96% accuracy, 0.993 AUC, and ECE = 0.0165) that provides a reliable foundation for the interpretability analysis that follows; (2) a decoder-assisted attribution method that introduces a novel reconstruction-decoder approach that circumvents resolution collapse at the network’s lowest-resolution bottleneck without requiring additional segmentation supervision; (3) systematic validation of this and standard attribution methods across four complementary quantitative metrics—faithfulness, spatial localization, progressive masking behavior, and inter-method agreement; and (4) the identification of a class-specific dissociation in attribution reliability between glioma and meningioma, demonstrating that standard shallow-to-mid-layer explainability approaches, though effective for one tumor type, can fail entirely for another within the same classification task.

The present work falls within the scope of AI-enabled sensing and data analytics, with particular relevance to multimodal data fusion and signal processing. Multi-parametric MRI is a volumetric imaging modality in which complementary contrasts (T1c, T1n, T2f, and T2w) are acquired by the scanner and must be processed jointly to yield clinically usable information. The proposed pipeline fuses the four contrasts in a single 3D deep learning classifier with calibrated confidence outputs, after a per-patient intensity normalization of the measured signal. Its explainability component can be viewed through a signal-processing lens. Successive downsampling in the encoder progressively discards fine-scale spatial detail, while the reconstruction decoder, through its skip connections, restores the detail from the coarse bottleneck. These elements address the reliability, interpretability, and data-homogeneity challenges that arise when AI models are applied to data from imaging systems [35,36].

## 2. Materials and Methods

### 2.1. Dataset

The dataset used in this experiment was taken from the BraTS 2023 challenge [37,38,39]. The data are structural magnetic resonance images, a biomedical imaging sensing modality in which the radiofrequency receiver coils of the MRI scanner record the signal emitted by tissue after excitation. The images are preoperative scans acquired with clinical scanners and protocols at multiple institutions, and no additional data were acquired for this study. Each examination comprises four acquisition sequences: T1-contrast enhanced (T1c), T1 native (T1n), T2-FLAIR (T2f), and T2-weighted (T2w), which were used as the four input channels of a single volumetric sensor input. Expert tumor segmentations provided with the challenge were used only for evaluating the attribution maps, not for training the classifier or the decoder.

The two classes considered were glioma and meningioma, which makes the problem a binary classification. The entire dataset includes 2251 cases; the training/validation split consists of 1801 cases (1001 glioma; 800 meningioma), and the remaining 450 cases (250 glioma; 200 meningioma) were held out during training and model selection and were used for final evaluation only. Model selection was done via 4-fold cross-validation on the training/validation dataset.

### 2.2. Preprocessing

BraTS 2023 volumes [37,38,39] are released with standard preprocessing already applied, including rigid co-registration of all four modalities to the SRI24 anatomical atlas [40], 1 mm^3^ isotropic resampling, and automated skull-stripping. Building on this, each modality volume (native resolution, 240 × 240 × 155) was independently resampled to a fixed input resolution of 128 × 128 × 128 voxels using trilinear interpolation. Intensities were then normalized per patient and per modality channel using z-score standardization, because MRI signal intensities are expressed in arbitrary units that depend on the scanner, the receiver coils, and the acquisition sequence, and therefore have no absolute scale across devices and sites. Because the BraTS 2023 cohort aggregates data from multiple contributing institutions, per-patient normalization also serves to partially harmonize potential site- and scanner-level acquisition differences between data collections. The four normalized modality volumes were then stacked along a channel dimension to form a single multimodal input tensor of shape [4, 128].

**Figure 1:**
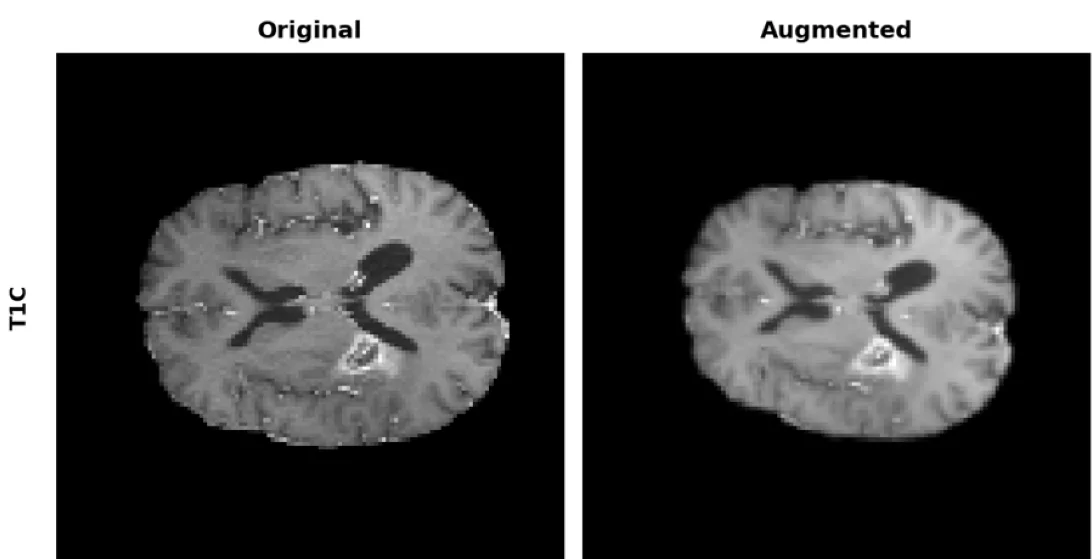
Example of data augmentation applied to a training sample. Original (**left**) and augmented (**right**) axial T1c slice from a glioma patient, illustrating the combined effect of the augmentation pipeline. This example primarily reflects rotation and intensity shift/scale augmentation.

**Figure 2:**
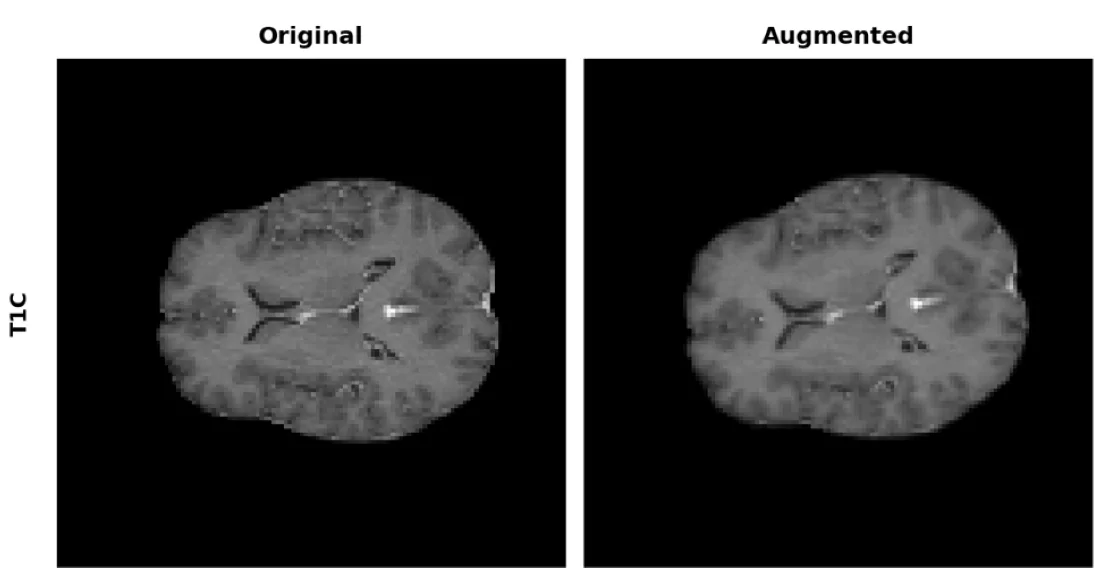
Example of data augmentation applied to a training sample. Original (**left**) and augmented (**right**) axial T1c slice from a meningioma patient, illustrating the combined effect of the augmentation pipeline. This example primarily reflects rotation and intensity shift/scale augmentation.

Since the BraTS 2023 cohort aggregates data from multiple contributing institutions, per-patient normalization also serves to partially harmonize potential site- and scanner-level acquisition differences between data collections. The four normalized modality volumes were then stacked along a channel dimension to form a single multimodal input tensor of shape [4, 128]. To improve generalization, given the limited size of medical imaging datasets, online data augmentation (random flip, ±10° axial rotation, intensity shift/scale, and zoom) was applied to the training set, with spatial transformations applied identically across all four modality channels to preserve cross-modal anatomical correspondence. Figure 1 and Figure 2 illustrate representative examples of the aforementioned augmentation.

### 2.3. Model Architecture

**Figure 3:**
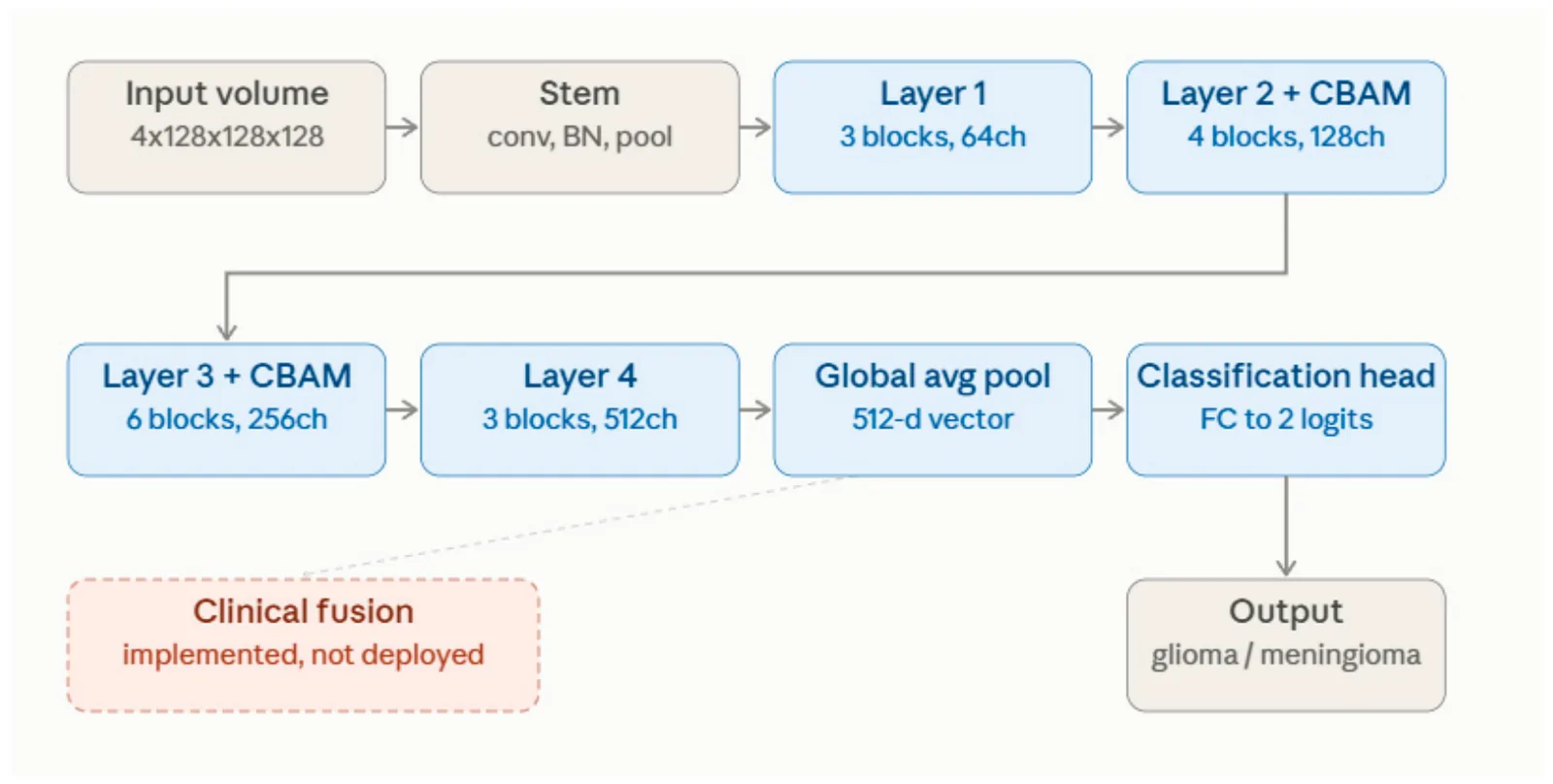
3D ResNet-34 backbone architecture with CBAM attention modules. The four residual stages (Layer 1–4) process the four-channel input volume sequentially, with CBAM attention integrated after Layer 2 and Layer 3. A clinical fusion pathway incorporating demographic variables was implemented but not activated for the classification results reported in this study.

The classification backbone was a 3D ResNet-34 architecture, selected for its balance between representational capacity and computational tractability in the volumetric, multimodal setting of this study, and for its compatibility with MedicalNet, which provides pretrained 3D weights specifically for ResNet-family architectures. Deeper variants (e.g., ResNet-50/101) substantially increase parameter count and memory requirements, a meaningful constraint when processing four-channel 128^3^ volumetric input, while shallower architectures risk insufficient representational capacity for the fine-grained tissue and boundary distinctions relevant to glioma–meningioma discrimination. The architecture was adapted from the standard 2D ResNet-34 to operate on volumetric input, initialized with pretrained weights from MedicalNet [41] to leverage transfer learning from large-scale 3D medical imaging data despite the limited size of the training set [42]. The network processes the four-channel input tensor [4, 128] through a series of residual stages, each comprising 3, 4, 6, and 3 BasicBlock3D units respectively, with 3D convolutions, batch normalization, and ReLU activations replacing their 2D counterparts throughout. Following the convention used in this study, these four residual stages are referred to as Layer 1 through 4 and should not be confused with individual convolutional layers. Figure 3 illustrates the complete architecture.

To enhance the network’s ability to focus on diagnostically relevant regions, Convolutional Block Attention Module (CBAM) blocks were integrated after the second and third Layer. CBAM sequentially applies channel and spatial attention [43]:

(1) ***M_c_***(x) = ***σ_sig_***(MLP(AvgPool(x)) + MLP(MaxPool(x)))

(2) ***x^′^*** = ***M_c_***(x) × (x)

where ***σ_sig_*** is the sigmoid function, and MLP is a shared two-layer bottleneck (reduction ratio: C → C/16 → C). The channel-refined feature map, x′, is then passed through spatial attention:

(3) ***M_s_***(***x^′^***) = ***σ_sig_*** (***W***_7×7×7_ [***AvgPool_c_***(x′); ***MaxPool_c_***(x′)])

(4) ***x^″^*** = ***M_s_***(x′) × (x′)

where average and max pooling are applied across the channel dimension, concatenated, and processed by a 3D convolution to produce a spatial attention map.

### 2.4. Training and Evaluation Configuration

Cross-entropy loss with label smoothing (ε = 0.1) was used in training to enable well-calibrated probabilistic predictions that are not overconfident [44]. As for the imbalance between the classes (1001 glioma; 800 meningioma), an inverse-frequency weighting strategy was used [45], penalizing the minority class (meningioma) more during the training process but not having to rely on resampling. Adam optimizer (η = 0.0001, ***β***_1_ = 0.9, and ***β***_2_ = 0.999) with 5-epoch linear warm-up was used [46], along with ReduceLROnPlateau scheduling (factor, 0.5; patience, 5 epochs on validation loss). Early stopping was applied after 15 epochs of no improvement on the validation accuracy (maximum of 150 epochs), keeping the checkpoint corresponding to the best performance on the validation set according to the highest accuracy. Moreover, gradient norm clipping was applied to stabilize training [47].

Training followed a 4-fold cross-validation protocol at the patient-level, with the best-performing fold’s checkpoint selected for a single, final evaluation on the held-out test set.

### 2.5. Explainability Pipeline

To provide spatially interpretable explanations of the network’s classification decisions, a progressive explainability pipeline was deployed across the four residual stages of the architecture (Layer 1–4). Standard Grad-CAM was first applied at each stage, using the gradient of the predicted class score with respect to the stage’s feature maps to produce a class-discriminative localization map. However, at deeper stages, where feature maps carry the most semantically relevant information for classification while having undergone substantial spatial downsampling, Grad-CAM’s coarse resolution proved insufficient to yield reliable explanations, particularly for meningioma, a class whose diagnostic signal often depends on global, boundary-adjacent context rather than a spatially compact region [48].

SmoothGrad was subsequently applied to reduce gradient noise by averaging saliency maps over multiple, noise-perturbed copies of the input. This improved the visual smoothness of the resulting maps, but it still did not resolve the underlying resolution collapse at Layer 4, where feature maps are reduced to a spatial resolution of 4 × 4 × 4 voxels (too coarse to be resampled back to the 128 × 128 × 128 input space without severe loss of spatial precision [49]).

**Figure 4:**
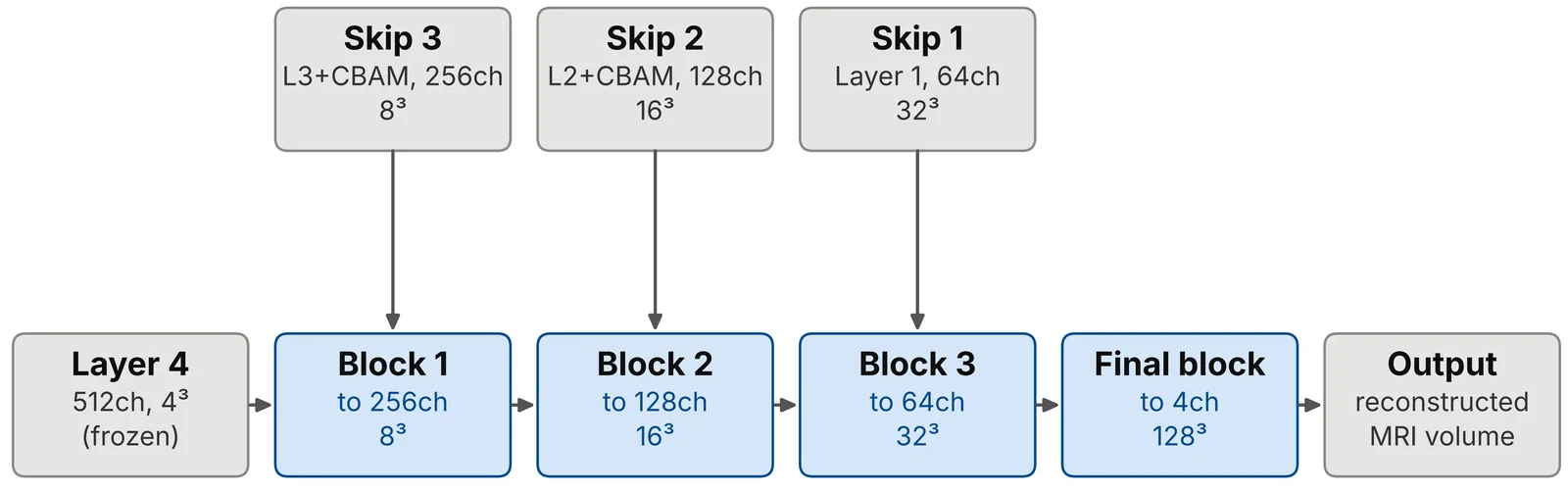
Decoder architecture diagram.

To resolve the resolution collapse at Layer 4, a novel reconstruction decoder, designed to recover spatially precise attribution without requiring segmentation supervision or additional annotation, was developed. A 3D decoder network was trained with mean squared error loss to reconstruct the original four-channel MRI volumes from Layer 4, using skip connections from Layers 1–3 to recover the spatial detail lost in the 4 × 4 × 4 bottleneck. Grad-CAM was then applied using the decoder’s mean output as the scalar backward target, allowing gradients to propagate through the full reconstruction pathway back to Layer 4 features, yielding attribution maps at full 128 × 128 × 128 resolution without the upsampling artifacts that make direct Layer-4 Grad-CAM uninformative. Channel weights were computed as the spatial mean of the gradients, the weighted sum of the feature maps was passed through a ReLU so that only features with a positive influence on the target score were retained, and the map was divided by its maximum value to give values in [0, 1]. Figure 4 illustrates the decoder architecture.

The decoder-assisted attribution is therefore decoder-mediated. Gradients of the reconstruction output are propagated back through the decoder, so the resulting map integrates the Layer 4 bottleneck with the shallower encoder features supplied through the skip connections. This construction is motivated by the bottleneck resolution at 4 × 4 × 4, where each cell spans 32 voxels per edge of the 128^3^ output, comparable to the extent of an average tumor, which limits the spatial precision attainable with Grad-CAM applied directly at Layer 4. The Decoder is made up of three upsampling blocks, along with a final output block. In each block, there is a process of trilinear upsampling in which the dimensions are made equal to those of skip connections, which then get concatenated; and finally, two Conv3D blocks, one BatchNorm3D block, and one ReLU block are used. The dimensionality of the channels decreases progressively, in the following manner: from 512 (Layer 4) to 256 (skip3, 8^3^), to 128 (skip2, 16^3^), and then to 64 (skip1, 32^3^). Further, another layer is created, where the channel dimension is decreased from 64 to 32 and then to 4, giving out an output of 4 channels and dimensions 128^3^.

For the optimization of the decoder network, the Adam optimizer was used (learning rate = 1 × 10^−4^; weight decay = 1 × 10^−5^) with ReduceLROnPlateau scheduler (patience = 3; factor = 0.5) and gradient norm clipping (max norm = 1.0). The minimization of the objective function entailed the use of the mean squared error (MSE) difference between the reconstructed and the actual inputs. The batch size was set to 2 for up to 30 epochs, with an early stopping after a patience of 7 epochs in respect to the validation loss. The split of 85/15 was applied for training/validation group (n = 1531/n = 270). The test set was held out during the decoder training.

Training was consistent throughout the entire duration of the 30 epochs, with the lowest validation MSE being 0.0521, and the maximum PSNR being 12.83 dB. However, while the reconstruction is lossy, it is adequate to serve the purpose of the decoder, which is to offer a full-resolution path for gradient flow instead of generating high-quality reconstructions that can be used for diagnosis.

For an input volume, the frozen encoder provides the Layer 1–3 skip features and the Layer 4 bottleneck features, which the frozen decoder maps to a four-channel reconstruction at 128^3^. The scalar backward target is the mean of the decoder output. Feature maps and the gradients of this target with respect to them are taken at the decoder’s final convolutional layer; the channel weights are the spatial averages of these gradients, and the raw attribution map is scaled to a maximum of 1. Layers 1–3 use standard Grad-CAM with the gradient of the predicted-class score.

An important distinction between the different computation layers (1–3) of the Grad CAM was noted, which is conditioned directly on the predicted class label, and the decoder-assisted attribution at Layer 4, which uses the decoder’s mean reconstruction score as its backward-pass target and therefore is not directly class-conditioned at the point of computation. Class-relevant structure is present in the underlying features that the decoder operates on: the Layer 4 bottleneck and the skip connections from Layers 1–3 (the latter two additionally refined by CBAM attention) are all outputs of the classification-trained encoder. The decoder provides a differentiable pathway through which this pre-existing, classification-derived signal can be propagated to full resolution, rather than introducing class-specific information on its own. This is further supported empirically in Section 3.2.3, where ablating the skip connections while retaining only the decoder’s reconstruction pathway causes the resulting attribution to lose all patient-specific spatial grounding.

Table 2 summarizes the explainability method and corresponding feature map resolution at each residual stage.

### 2.6. Explainability Validation Metrics

Four complementary quantitative metrics were used to validate attribution quality across all four residual stages, chosen to jointly assess faithfulness, spatial localization precision, progressive masking behavior, and inter-method spatial agreement. The faithfulness, pointing-game, and Spearman analyses were evaluated on the best-prediction subset (n = 420), consisting of test cases that were both correctly classified and predicted with confidence exceeding 0.9. Test cases were grouped by prediction correctness and confidence: correct predictions with confidence above 0.9 (best-prediction subset, n = 420), incorrect predictions with confidence above 0.9 (confident errors, n = 15), and the remaining cases with intermediate confidence (0.7–0.9, n = 15). The error cases and their confidence distribution are described in Section 3.4.2. This restriction isolates attribution quality from classification uncertainty. Insertion/deletion curves were evaluated on the complete test set (n = 450). For completeness, the faithfulness, pointing-game, and Spearman analyses were also computed on the complete test set (n = 450) and are reported in Table A2, Table A4 and Table A8.

Faithfulness/perturbation test. For each patient, the top 10% of most activated voxels were replaced with per-channel mean values, representing a masking strategy suited to MRI data, where zero-masking would introduce out-of-distribution intensities. The resulting confidence drop was compared to random masking of the same number of voxels (averaged over five random masks), yielding a faithfulness delta: a positive delta indicates the highlighted region genuinely drove the model’s confidence [50]. This mean-masking approach was confirmed in subsequent ablation analyses (Section 3.2.3), which produced numerically consistent results under an alternative mean-fill masking convention applied to internal network representations.

Pointing game for spatial localization precision. For each patient, the single voxel of maximum attribution value across the full 128^3^ heatmap was checked against the ground-truth BraTS tumor segmentation mask; a hit was recorded if it fell within the mask. The random baseline was the mean tumor volume relative to total volume (~1.8%) [51].

Insertion/deletion curves. While the perturbation test is a faithfulness evaluation at one specific threshold (top 10% of attributed voxels), the insertion and deletion curves test faithfulness at a continuous range of masking fractions, allowing us to observe the evolution of confidence rather than measure its value at some arbitrarily chosen points. It solves the issue of a model not performing well at 10% masking while being inconsistent with different thresholds, because the curve is much less affected by a particular threshold artifact than an evaluation at a single point. Model confidence was evaluated across a range of masking fractions (1%, 2%, 5%, 10%, 20%, 30%, 50%, 70%, and 100% of voxels). For deletion, voxels were progressively masked in decreasing attribution order, expecting fast confidence reduction (low AUC). For insertion, from a fully masked image, a faithful method would have fast confidence recovery (high AUC) [52].

Spearman rank correlation. Pairwise Spearman rank correlation, ρ, was computed between flattened heatmap vectors for all six method-pair combinations across the 420 best-prediction patients, assessing monotonic spatial agreement between methods without assuming linear relationships, evaluated overall and separately per tumor class.

## 3. Results

### 3.1. Classification Performance

The mean validation accuracy and AUC after applying 4-fold cross-validation to the dataset were 98.33% ± 0.58 and 0.992 ± 0.005 [53], respectively (Table 3). The highest validation accuracy and AUC of 98.89% and 0.998 were observed in fold 2, which was used as the basis of selecting the model for evaluation on the independent test set.

In the case of the test data (n = 450), the accuracy of the classification achieved by the model was 96%, and the AUC was 0.993. In terms of per-class metrics, the precision and recall scores achieved by the model for glioma were 0.99 and 0.94, whereas those of meningioma were 0.92 and 0.985, with F1-scores of 0.96 and 0.95, respectively. In terms of specificity, the values were 0.985 for glioma and 0.94 for meningioma (Table 4).

**Figure 5:**
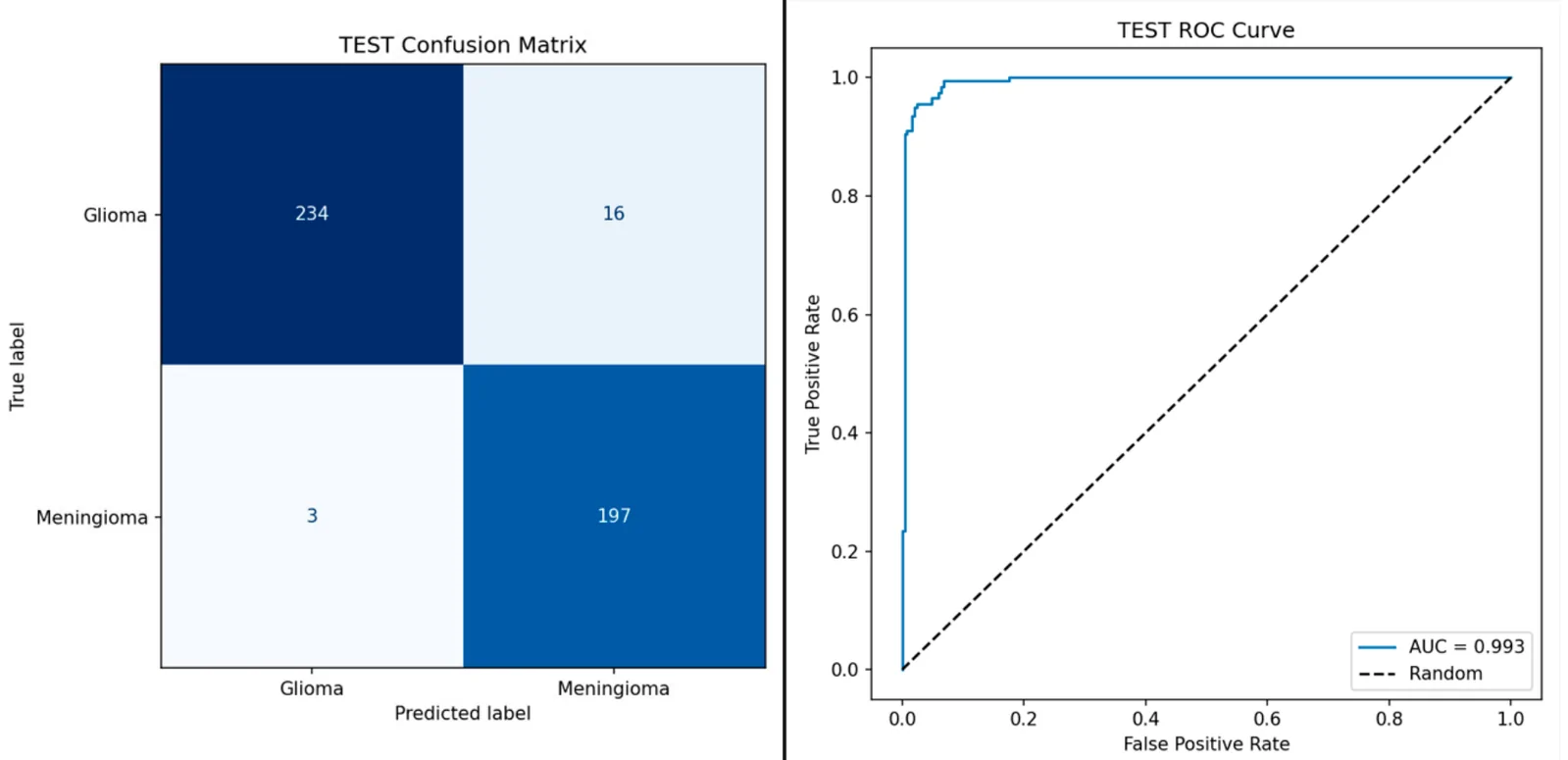
Test set classification performance: (**Left**) Confusion matrix showing true vs. predicted labels for glioma and meningioma; and (**right**) ROC curve with corresponding AUC (0.993).

The confusion matrix and ROC graph of Figure 5 indicate that 234 out of 250 glioma samples and 197 out of 200 meningioma samples are correctly classified, but the classification error appears in the form that 16 samples are classified as meningioma and three meningiomas as glioma. This imbalance is quite similar to the one noticed from the higher recall value of meningioma samples, which is 0.985 compared to the glioma, which is 0.94 (Table 4).

**Figure 6:**
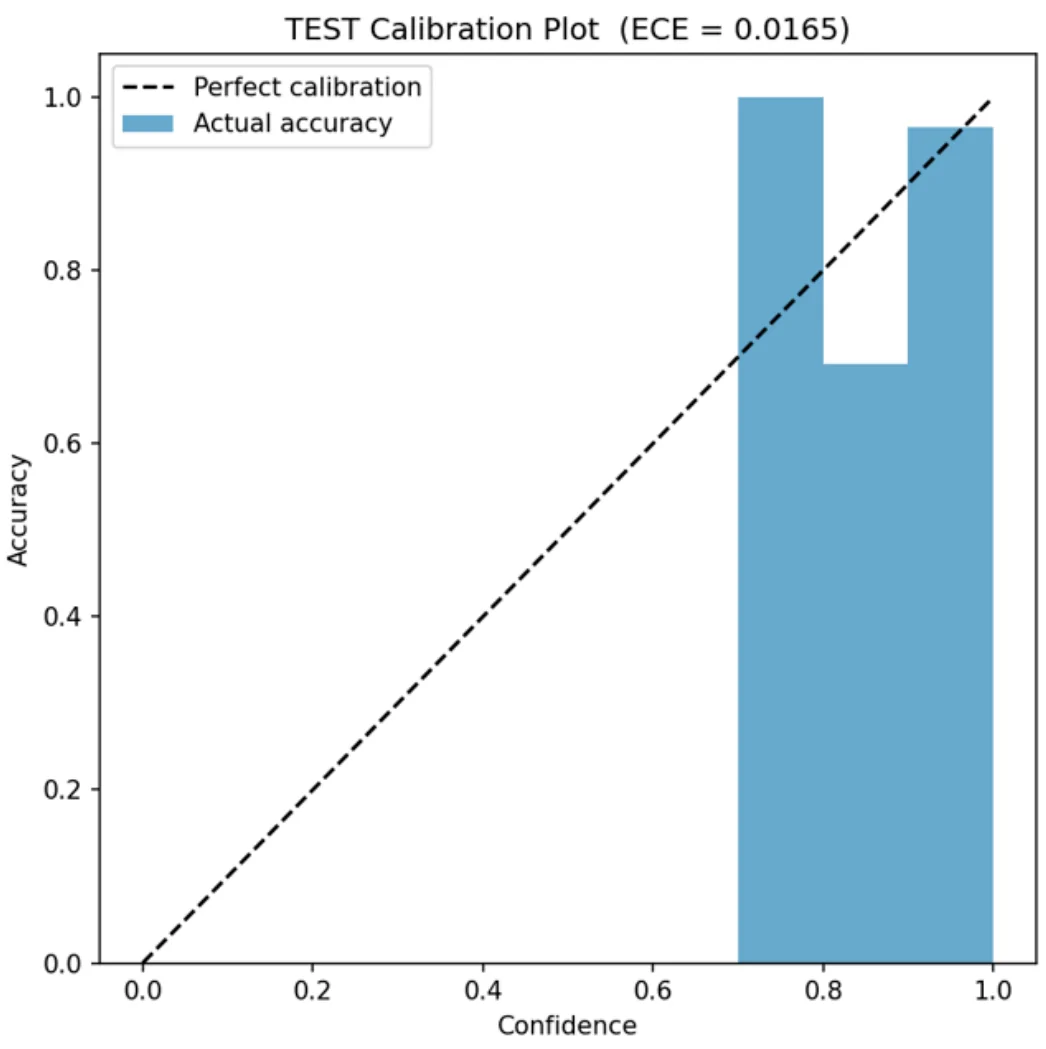
Calibration plot of the test set, comparing predicted confidence against empirical accuracy (ECE = 0.0165). The dashed diagonal represents perfect calibration.

Model calibration was assessed via expected calibration error (ECE) on the test set, yielding ECE = 0.0165 (Figure 6), indicating close alignment between predicted confidence and empirical accuracy. This confirms the effectiveness of label smoothing in producing well-calibrated probability estimates, a property particularly relevant for the confidence-stratified attribution analysis [44,45]. As an additional measure of calibration, Brier score, the average squared difference between the predicted probability and the actual class, was calculated at 0.0397, meaning that the method has great probabilistic calibration. The class-wise expected calibration error (ECE) was calculated as 0.0100 for gliomas and 0.0246 for meningiomas, meaning a somewhat worse calibration for meningiomas in comparison with gliomas. Resampling through bootstrapping technique with 1000 iterations gave a 95% confidence interval for accuracy of [93.78%, 97.56%]; for AUC, of [0.985, 0.998]; and for ECE, of [0.005, 0.034].

### 3.2. Explainability Validation Results

To assess whether the attribution methods described in Section 2.5 genuinely reflect the model’s decision-making process, results are presented across five complementary analyses: representative attribution heatmaps, a faithfulness/perturbation test, a pointing-game evaluation, insertion/deletion curves, and pairwise Spearman rank correlation between methods. Together, these analyses move from qualitative visual inspection to increasingly rigorous quantitative validation, converging on a consistent finding: a class-specific dissociation in the spatial scale of features driving glioma versus meningioma classification, discussed further in Section 3.4.

#### 3.2.1. Attribution Heatmaps Across Layers

**Figure 7:**
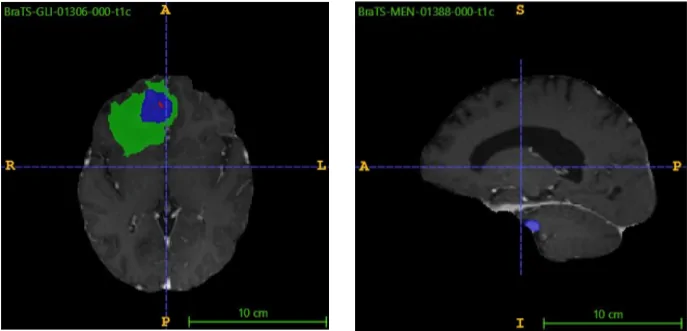
Ground-truth tumor location for the representative glioma and meningioma patients shown in Figure 8 and Figure 9. (**Left**) Axial T1c slice of the glioma patient with BraTS 2023 segmentation overlay (green, peritumoral edema; blue, non-enhancing tumor core; and red, enhancing tumor). (**Right**) Sagittal T1c slice of the meningioma patient with BraTS 2023 segmentation overlay (blue, meningioma tumor mass).

**Figure 8:**
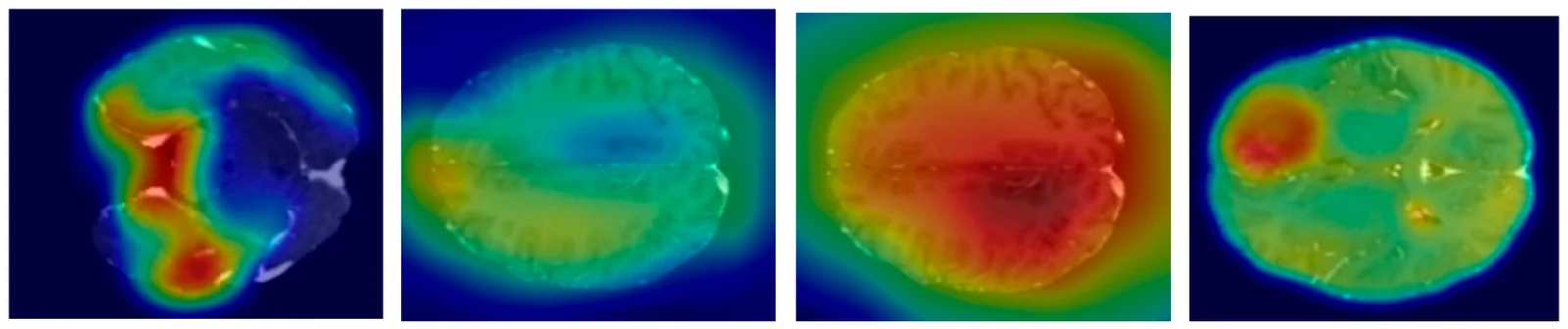
Attribution heatmaps across residual stages (Layer 1–4) for a representative glioma patient, axial T1c slice. Warm colors indicate high attribution; cool colors indicate low attribution. Attribution progressively localizes onto the tumor mass, with the decoder-assisted Layer 4 attribution achieving the tightest focal concentration. The underlying MRI volume is identical across all four layers in each figure; the displayed axial slice is independently selected per layer as the slice of maximum summed attribution, since peak activation location can shift across network depth.

**Figure 9:**
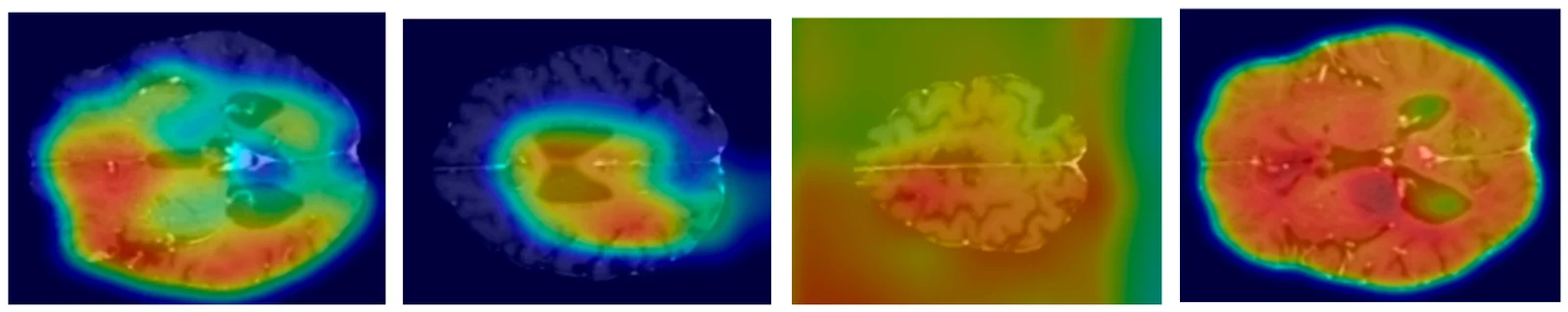
Attribution heatmaps across residual stages (Layer 1–4) for a representative meningioma patient, axial T1c slice. Warm colors indicate high attribution; cool colors indicate low attribution. Attribution remains diffuse or spatially uncorrelated with the tumor location through Layer 3, with the decoder-assisted Layer 4 attribution converging on the region surrounding, rather than internal to, the tumor mass.

Figure 7 shows the ground-truth tumor location for a representative glioma patient (axial T1c) and meningioma patient (sagittal T1c), based on BraTS 2023 segmentation annotations, providing an anatomical reference against which the attribution heatmaps in Figure 8 and Figure 9 can be evaluated.

Figure 8 and Figure 9 present representative attribution heatmaps for a correctly classified glioma patient and a correctly classified meningioma patient (confidence > 0.9), respectively, across all four residual stages, overlaid on the corresponding MRI. At Layer 1 (32^3^), attribution for both classes is broad and diffuse, reflecting the model’s early-stage sensitivity to low-level intensity and geometric patterns rather than tumor-specific features; for meningioma, activation is already weighted toward the peripheral, extra-axial space, an early hint of the global spatial reasoning that becomes more pronounced at deeper layers. At Layer 2 (16^3^), glioma attribution begins concentrating more tightly within the brain interior, while meningioma attribution retains near-zero spatial specificity to the tumor region. At Layer 3 (8^3^), glioma attribution shows coherent, progressively tighter localization, whereas meningioma attribution becomes spatially uncorrelated with the tumor location entirely, consistent with the near-zero faithfulness and Spearman agreement reported in Section 3.2.2, indicating that this intermediate depth captures neither the broad early-layer context nor a coherent higher-level spatial signal for meningioma [54].

At Layer 4, the decoder-assisted attribution reveals the central visual finding of this study: a class-specific dissociation in spatial reasoning. For glioma, attribution converges tightly onto the tumor mass with minimal spread into surrounding tissue, the sharpest localization achieved anywhere in the pipeline. For meningioma, instead, attribution distributes across the extra-axial space and brain-skull interface surrounding the tumor, rather than the tumor mass itself. This pattern is consistent with general anatomical distinctions between the two tumor types, glioma as an intra-axial lesion and meningioma as an extra-axial mass [55].

#### 3.2.2. Quantitative Validation Metrics

Faithfulness was assessed via a perturbation test, comparing model confidence drop after masking the top 10% attributed voxels against random masking; all four methods produced positive mean deltas, confirming no produce of artifactual attribution. Spatial localization precision was assessed via the pointing game, checking whether the single highest-attribution voxel fell within the ground-truth tumor mask (random baseline, ~1.8%); Layer 4 substantially outperformed all other layers (80.2% vs. 6.4–21.9%). Insertion/deletion curves extended this assessment across a continuous range of masking fractions rather than one threshold, with Layer 4 achieving the lowest deletion AUC (0.506) of any method, indicating the most rapid confidence loss when its highlighted regions were removed. Layer 1 reached the highest insertion AUC (0.919 vs. 0.887 for Layer 4), and Layer 2, the highest mean faithfulness delta; these metrics reward broader or threshold-based attribution, whereas the pointing game and deletion AUC emphasize precise localization, where the decoder-assisted Layer 4 performed best. Pairwise Spearman rank correlation between methods showed Layer 1 and the decoder as the most closely aligned pair (ρ = 0.846), while Layer 3 showed the weakest agreement of all other methods. To assess whether these findings depend on the restriction to high-confidence correct predictions, faithfulness, pointing-game, and Spearman analyses were additionally computed on the complete test set (n = 450; Table A2, Table A4 and Table A8). The layer-wise pattern was preserved, and pointing-game accuracy of the decoder-assisted Layer 4 was unchanged (80.2%). The remaining 30 cases, which include confident misclassifications, showed lower faithfulness. Table 5 summarizes the above quantitative methods.

**Table 5:** Summary of quantitative XAI validation metrics across residual stages. Insertion/deletion values are computed on all 450 test patients. All other metrics on the best-prediction subset (n = 420).

Method	Faithfulness (Overall Δ)	Pointing Accuracy	Deletion AUC	Insertion AUC	Spearman vs. Decoder
Layer 1	+0.233	21.9%	0.584	0.919	0.846
Layer 2	+0.291	6.4%	0.616	0.851	0.565
Layer 3	+0.0.72	13.1%	0.620	0.655	0.357
Layer 4	+0.217	80.2%	0.506	0.887	n/a

#### 3.2.3. Contribution of Layer 4 and Skip Connections

To isolate the individual contributions of the Layer 4 bottleneck and the skip connections to decoder-assisted attribution, pointing-game accuracy and faithfulness were recomputed on the best-prediction subset (n = 420), with each component replaced by zero values (and, as a robustness check, by its own per-channel spatial mean) prior to decoder-assisted Grad-CAM computation. The results are summarized in Table 6.

**Table 6:** Pointing-game accuracy and faithfulness delta under ablated conditions.

Condition	Pointing (Ovr)	Pointing (Glioma)	Pointing (Meningioma)	Faithfulness (Overall)	Faithfulness (Glioma)	Faithfulness (Meningioma)
Baseline	80.2%	91.2%	67.4%	0.217	0.139	0.309
Layer 4 zeroed	71.7%	79.7%	62.2%	0.396	0.006	0.856
All skips zeroed	0.0%	0.0%	0.0%	−0.013	0.004	−0.033
Skip1 only zeroed	8.6%	9.3%	7.8%	0.288	−0.001	0.628
Skip2 only zeroed	73.1%	78.4%	66.8%	—	—	—
Skip3 only zeroed	71.9%	79.7%	62.7%	—	—	—

Removing all skip connections collapsed the pointing-game accuracy to 0% and faithfulness to near-zero, indicating that Layer 4 alone is insufficient to produce patient-specific attribution. Layer 4 lowered pointing-game accuracy from 80.2% to 71.7% (glioma, 91.2% to 79.7%; meningioma, 67.4% to 62.2%), indicating that localization is carried mainly by the high-resolution skip pathway, with the bottleneck supplying the deepest encoder features to the decoder.

Among individual skip connections, skip1 (the highest-resolution skip, from Layer 1) accounted for nearly all of this effect; removing skip2 or skip3 alone had minimal impact. Removing Layer 4 alone produced a moderate decline in glioma performance but a substantial increase in meningioma faithfulness (0.309 to 0.856), suggesting that skip-connection-driven attribution may capture extra-axial features relevant to meningioma classification that fall outside the strict tumor mask used by the pointing-game metric (Section 2.6). Mean-fill ablation reproduced these results almost exactly, confirming they reflect genuine loss of spatial information rather than an artifact of the replacement value used.

#### 3.2.4. Adversarial Robustness

Classifier and attribution robustness were assessed using the Fast Gradient Sign Method (FGSM) at three perturbation magnitudes (ε = 0.01, 0.03, and 0.05), applied to the z-score normalized input. A random-noise control of identical magnitude, with the perturbation direction chosen randomly rather than adversarially, was run for comparison. Table 7 summarizes the results.

**Table 7:** Adversarial robustness results.

Epsilon	Flip Rate (Ovr)	Flip Rate (Glioma)	Flip Rate (Meningioma)	Attribution Spearman
0.01	6.9%	5.7%	8.3%	0.757 (n = 391)
0.03	56.7%	33.9%	83.4%	0.757 (n = 182)
0.05	74.3%	53.7%	98.5%	0.759 (n = 108)
Random-noise control (all ε)	0.0%	0.0%	0.0%	—

Classifier predictions showed a class-specific difference in sensitivity to adversarial perturbation, with meningioma predictions altered far more readily than glioma at the same magnitude. The random-noise control produced no flips at any magnitude, indicating this sensitivity is specific to the deliberately optimized adversarial direction rather than a general instability; predictions remained fully stable under perturbations of identical magnitude applied in random directions. Attribution stability among unflipped cases remained consistent (Spearman ρ ≈ 0.76) at ε = 0.01, where sample sizes were still large; this estimate is not well-supported at higher ε values due to shrinking sample size among surviving meningioma cases.

### 3.3. Relationship Between Glioma and Meningioma Attribution Results

Across the four XAI validation metrics reported in Section 3.2.2, a consistent pattern is observed: Layers 1–3 perform well for glioma but poorly for meningioma, while the decoder-assisted Layer 4 attribution performs well for both classes. For glioma, faithfulness, pointing accuracy, and inter-method agreement remain consistently high across nearly all layers (e.g., 98–100% faithfulness and ρ ≥ 0.606 for all Layer 1–3 method pairs). For meningioma, Layers 1–3 show near-zero or negative faithfulness, low pointing accuracy, and near-zero or negative Spearman correlation at Layer 3, while the decoder-assisted attribution achieves 96.9% faithfulness, 67.4% pointing accuracy, and 0.926 insertion AUC (n = 450). This pattern is consistent across all four independently designed metrics.

Layer 4 achieves the highest pointing accuracy and the lowest deletion AUC, whereas Layer 1 has the highest insertion AUC, and Layer 2, the highest overall faithfulness delta (driven by glioma). Layer 4 is the only layer with a clearly positive faithfulness delta for both classes. It achieves the highest overall pointing accuracy (80.2%, ~45× random baseline), the lowest overall deletion AUC (0.506), and the strongest agreement with Layer 1 of any method pair (ρ = 0.846). It is the only method that performs well on the faithfulness, localization, and insertion/deletion metrics for both tumor classes. As localization depends mainly on the high-resolution skip pathway (Section 3.2.3), these results characterize the decoder-mediated attribution.

### 3.4. Error Case Analysis

#### 3.4.1. Representative Misclassified Cases

**Figure 10:**
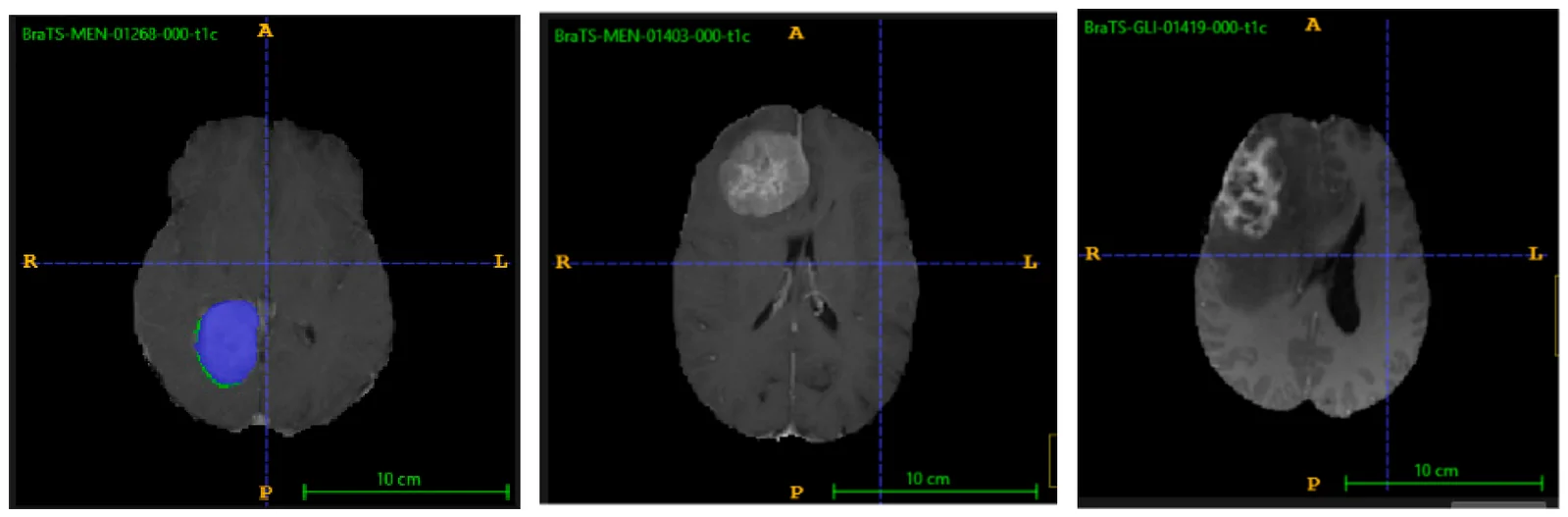
Representative misclassified test cases. (**Left**, **center**) Two meningioma cases predicted as glioma, both presenting as large, intra-axial-appearing masses without characteristic extra-axial features. (**Right**) A glioma case predicted as meningioma, presenting as a large mass attached along the brain’s outer border.

Of the 19 test-set misclassifications, 16 were glioma cases predicted as meningioma, and three were meningioma cases predicted as glioma, consistent with the higher recall observed for meningioma relative to glioma (Section 3.1). Figure 10 presents three representative misclassified cases. Two meningioma cases predicted as glioma both presented as large, intra-axial-appearing masses without the well-circumscribed, extra-axial characteristics typical in meningioma, visually resembling glioma even prior to model prediction. The glioma case predicted as meningioma was also large in extent, but attached along the brain’s outer border, a presentation more characteristic of meningioma’s typical extra-axial position. These cases suggest that at least a subset of the model’s errors corresponds to atypical tumor presentations that diverge from each class’s characteristic imaging appearance, rather than representing arbitrary model failures.

#### 3.4.2. Prediction Confidence of Misclassified Cases

**Figure 11:**
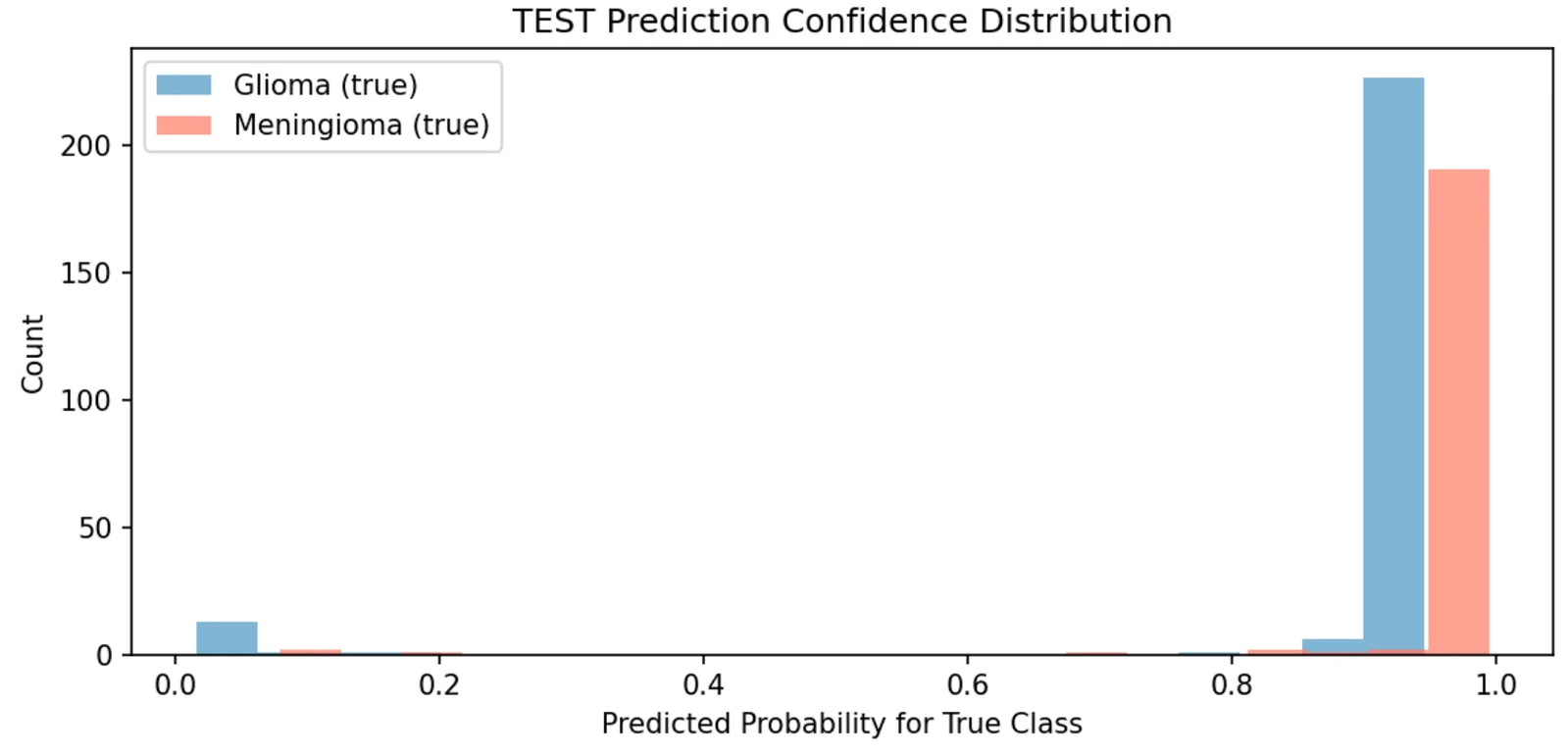
Distribution of predicted probability for the true class on the test set, by tumor type. Values near 1.0 indicate high-confidence correct predictions, while values near 0 correspond to high-confidence errors (a low predicted probability for the true class means the model was confident in the wrong class).

Of the 19 misclassification errors on the test set, the majority (15/19, 79%) were confident errors, predictions made with confidence exceeding 0.9 despite being incorrect—while no errors occurred in the uncertain regime (confidence < 0.7); the remaining four errors fell in an intermediate confidence range (0.7–0.9) (Figure 11). This pattern indicates that the model’s overall low ECE (0.0165, Section 3.1) does not preclude a specific and clinically important failure mode: when the model is wrong, it is usually wrong with high confidence rather than appropriately uncertain, meaning confidence alone cannot reliably flag these cases for clinician review. Because 435 of the 450 test predictions (97%) have confidence above 0.9, most errors necessarily fall in this range; accuracy was 96.6% (420/435) for predictions above 0.9, and 73% (11/15) for those between 0.7 and 0.9.

#### 3.4.3. Attribution of Misclassified Cases

**Figure 12:**
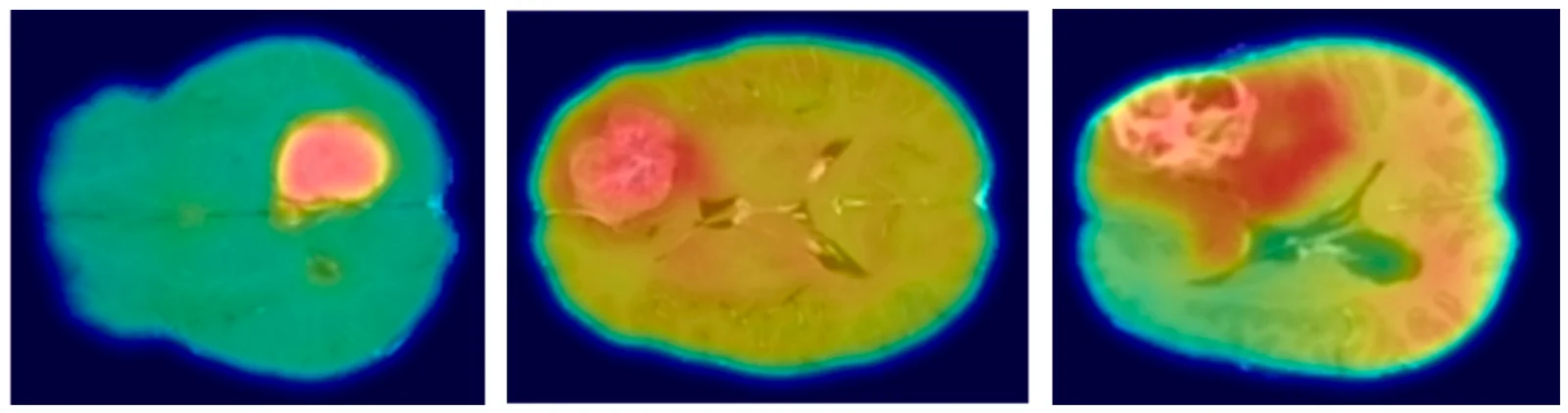
Decoder-assisted Layer 4 attribution for the same three misclassified cases shown in Figure 10. (**Left**, **center**) The two meningioma cases predicted as glioma; (**right**) the glioma case predicted as meningioma. Red indicates the highest attribution (regions contributing most to the decoder-assisted score) and blue the lowest.

To assess whether these confident errors reflect genuinely ambiguous cases or model failures unrelated to case difficulty, a subset of misclassified cases was reviewed by practicing clinicians. Based on this review, some misclassified cases presented genuinely ambiguous or atypical imaging features that plausibly account for the model’s error, consistent with known overlap in imaging presentation between glioma and meningioma (Figure 12). Other cases showed no such clinical justification, suggesting a failure of the model’s learned representation rather than case difficulty. This review was conducted on a limited case subset without blinding to the model’s prediction, and we treat it as a preliminary clinical observation rather than a systematic validation study; formal, blinded radiological validation across the full error set is identified as a direction for future work.

## 4. Discussion

### 4.1. The Reconstruction Decoder as a Methodological Contribution for XAI

This study contributes to two complementary fronts. First, the 3D multimodal classification pipeline itself, combining a MedicalNet-pretrained 3D ResNet-34 backbone with CBAM attention across four MRI modalities, provides an anatomically faithful, volumetric alternative to the 2D slice-based approaches that dominate the glioma–meningioma classification literature, achieving strong and well-calibrated performance (Section 3.1) that serves as a reliable substrate for the explainability analysis that follows. Second, and more centrally to this study’s aim, the reconstruction decoder introduced here addresses a limitation that extends beyond this particular classification task. Standard Grad-CAM-style attribution methods degrade at the deepest layers of a 3D CNN, precisely where the most class-discriminative features reside, because spatial resolution has typically collapsed to a small bottleneck (4^3^ in this architecture) that cannot be meaningfully resampled back to input resolution. Existing approaches to this problem generally require either sacrificing depth, aggregating or selecting attribution from shallower, less semantically informative layers, as in LayerCAM [56] and related multi-layer aggregation methods, or introducing additional supervision, such as segmentation masks or attention-guidance losses during training, as in guided attention inference approaches [57], to steer attribution toward relevant regions.

The relation of this approach to resolution recovery strategies in other fields is summarized in the Introduction. The decoder proposed here avoids both trade-offs: it is trained solely to reconstruct the input MRI volume, requiring no additional annotation beyond what is already used for classification, and it enables gradient-based attribution to propagate through the full reconstruction pathway from the Layer 4 and the skip features to full spatial resolution. This makes it an annotation-free strategy that may be of interest for other 3D CNN architectures facing the same resolution collapse problem. However, it is demonstrated here only for glioma and meningioma classification. This positions the present work at an intersection largely unoccupied in the existing literature: as explained in detail in Section 1, 3D volumetric classification of brain tumors remains comparatively rare, and where explainability has been incorporated into prior glioma–meningioma or related classification studies, validation has generally relied on qualitative or expert-agreement-based assessment rather than the quantitative faithfulness, localization, and inter-method agreement metrics applied here.

### 4.2. Interpretation of the Glioma–Meningioma Dissociation

The consistent pattern observed across all four XAI validation metrics (Section 3.2.2)—reliable attribution for glioma across nearly all layers, but, for meningioma, only once the decoder-assisted Layer 4 approach is applied—suggests that the two tumor types are not equally well represented by any single intermediate layer of the network, and that meningioma’s classification-relevant features become spatially resolvable only once the full hierarchical representation is reconstructed. This is consistent with a broader observation in the attribution literature: standard Grad-CAM-style methods are typically evaluated and shown to succeed on tasks where the relevant feature is spatially compact, and our results suggest this assumption may not hold uniformly across all classes within a single task, even when overall classification performance is high for both [58,59,60].

One plausible anatomical correspondence is that glioma, as an intra-axial lesion, may present a more spatially confined signal detectable at shallow depths, while meningioma, as an extra-axial mass defined partly by its relationship to surrounding structures, may require broader spatial context that only the full network depth captures.

### 4.3. Preliminary Clinical Review of Attention Patterns

To assess whether the network’s attention behavior corresponds to established clinical reasoning, a subset of the erroneous test cases was selected for clinical review. Cases in which the model’s misclassification could be readily explained by clear, unambiguous morphological features of the true tumor class, and were therefore judged likely attributable to model error rather than genuine diagnostic ambiguity, were excluded from this subset. The remaining cases, considered potentially atypical or diagnostically challenging, were presented to clinicians, who were shown the original MRI sequences alongside the true and predicted class for each case, and were asked to assess whether the imaging findings could plausibly account for the misclassification.

Clinical feedback on this subset was qualitative in nature. Atypical presentation was proposed as a possible contributing factor for some of the cases reviewed, while, for others, no clear radiological basis could be identified to distinguish them from straightforward misclassifications. Taken together, this suggests that the relationship between atypical tumor appearance and model error is complex, and further, that a more systematic clinical validation would help clarify it.

Clinical interpretation was also sought regarding selected Layer 4 decoder-assisted Grad-CAM visualizations, illustrating the local-versus-global attention pattern described in Section 4.2, wherein the model’s predictions for glioma are predominantly supported by local morphological features, while predictions for meningioma rely more heavily on broader positional context. This pattern was confirmed to reflect a standard diagnostic technique applied in practice for both tumor types: attending to local lesion morphology and to the lesion’s broader spatial context relative to surrounding structures. This indicates that the network’s attention behavior is consistent with established diagnostic reasoning across both classes. This correspondence lends qualitative support to the validity of the attribution patterns identified in Section 4.2, reinforcing that the observed local/global dissociation in the model’s attention, supported by both the quantitative validation metrics in Section 3.2.2 and this qualitative clinical review, mirrors a diagnostic strategy broadly consistent with clinical practice.

### 4.4. Discussion of Normalization Bias

Preprocessing applied per-patient, per-channel z-score normalization (Section 2.2), a standard and computationally efficient approach widely used in MRI analysis, and one that accounts for the scanner- and acquisition-dependent nature of MRI intensity values, given that intensity values across different scanners and acquisition protocols carry no fixed physical meaning. As with any normalization choice, alternative strategies exist and may offer complementary benefits, and the choice made here reflects a balance between methodological simplicity and practical efficiency rather than an assumption that it is the only reasonable approach. One such alternative would compute normalization statistics from healthy tissue alone, excluding the tumor region, potentially offering a more conservative standardization in settings where tumor size or composition varies substantially between classes. Such an approach may be particularly relevant in multi-institutional datasets, where subtle differences in tissue statistics between patient groups could, in principle, arise from a combination of biological and acquisition-related sources.

To characterize this further, post-normalization intensity statistics of healthy tissue, excluding a margin around the tumor, were compared between the glioma and meningioma cohorts in the training/validation data. Cohen’s d values were 0.49 (T1c), 0.71 (T1n), 1.15 (T2f), and 0.91 (T2w) in the training/validation data and 0.39, 0.59, 1.14, and 0.83 in the held-out test cohort, and the procedure was checked with a label-shuffle control (Appendix B). The intensity characteristics of the two cohorts can reflect several sources, including the extent of inflammation and tissue changes in the two classes, and characterizing their relative contributions would require acquisition-level information beyond that provided with the dataset. The evaluation reported in Section 3.1 and Section 3.2 was performed on a held-out test set that was not used for training or model selection. As a direct test of whether the normalization could carry class information, the per-patient normalization parameters alone were used to predict tumor class; the test-set AUC was close to 0.5 (0.51), whereas lesion volume alone gave 0.81 (Appendix B). This addresses the normalization constants only. Whether these cohort-level intensity characteristics or other information outside the tumor contributes to the learned decision rule cannot be excluded by the analyses presented, and because glioma and meningioma cases in BraTS 2023 come from separate challenge datasets and no site labels are available, class and acquisition site cannot be fully disentangled here. The online augmentation applied during training (Section 2.2), which included intensity shift and scale and zoom, exposes the network to variation in global intensity level and apparent size and may attenuate its reliance on absolute intensity values and size cues; however, this effect was not established here.

### 4.5. Limitations

Several caveats should be noted when analyzing the results presented. Firstly, the clinical review conducted in Section 4.3 was qualitative, non-blinded, and limited to a small subset of erroneous test cases; while it provided valuable preliminary insight supporting the link between the attributions found and the tumor features discussed in Section 4.2, a larger-scale, blinded, and systematically structured validation study would be needed to substantiate this correspondence with greater rigor, and is identified as a direction for future work (Section 4.6). Secondly, the data are drawn from the multi-center BraTS 2023 cohort, and per-patient intensity normalization was applied uniformly across all images. Healthy-tissue intensity statistics of the glioma and meningioma cohorts were characterized descriptively (Cohen’s d of 0.39–1.15 across modalities and cohorts; Section 4.4, Appendix B), and the normalization parameters alone gave a test AUC close to 0.5 (Appendix B). The contribution of other cohort-level or acquisition-related factors to classification was not evaluated and cannot be excluded; a tumor-masked evaluation is not reported here, because masking retains the size, outline, and position of the lesion, which are, themselves, informative about class, and external validation on data with documented acquisition sites would help verify generalization. Thirdly, the faithfulness, pointing-game and Spearman analyses in the main text were performed on the best-prediction subset (n = 420), while supplementary analyses on the complete test set (n = 450; Table A2, Table A4 and Table A8) showed a consistent layer-wise pattern, with lower values for the remaining cases.

Additionally, the decoder-assisted attribution at Layer 4 is derived from the bottleneck features through the decoder path, with its spatial detail derived mainly from the highest-resolution skip connection (Section 3.2.3), whereas the attributions at Layers 1–3 follow the standard Grad-CAM formulation; Section 3.2.3 supports the interpretation that class-relevant signal enters through the classification-trained encoder features underlying the decoder’s input, and further refinement of the attribution at the bottleneck represents a direction for future work (Section 4.6). Finally, adversarial robustness testing (Section 3.2.4) revealed that the classifier’s meningioma predictions are substantially more sensitive to small, deliberately engineered perturbations than glioma predictions; a random-noise control confirmed this sensitivity is specific to adversarially optimized perturbations rather than general instability under realistic input variation.

### 4.6. Future Work

First, an ablation study retraining the classification model using tumor-excluded normalization statistics would help quantify the relative contribution of genuine biological signal versus residual acquisition-related batch effects to the normalization bias finding in Section 4.4. Second, external validation on independently acquired MRI data with documented acquisition sites, from institutions or scanners not represented in BraTS 2023, would help establish whether both the classification performance and the decoder-assisted attribution approach generalize beyond the present dataset. Third, Ensembling across cross-validation folds, rather than selecting a single best-performing fold, represents an avenue for further improving classification robustness. Fourth, formalizing the confidence-based error flagging, demonstrated in Section 3.4.2, into an explicit “neither” or abstention output category would extend the present uncertainty-handling approach into a structural model capability. Fifth, a larger-scale, blinded, and systematically structured clinical validation study, extending beyond the preliminary qualitative review in Section 4.3, would help substantiate the observed correspondence between model attention and established clinical reasoning with greater methodological rigor. Finally, further refinement of the decoder-assisted attribution at the bottleneck represents an additional direction for future work.

## 5. Conclusions

This study presented an end-to-end pipeline for 3D CNN-based brain tumor classification, integrating a novel reconstruction-decoder approach to obtain full-resolution attribution where deep-layer resolution collapses. Using a 3D ResNet-34 with CBAM attention, trained on BraTS 2023 multimodal MRI (n = 2251), the model achieved strong classification performance (96% accuracy; 0.993 AUC) with well-calibrated confidence estimates (ECE = 0.0165), which were then rigorously validated across four complementary explainability metrics. The four metrics gave complementary results. Decoder-assisted attribution achieved the highest pointing-game accuracy (80.2%) and the lowest deletion AUC. Layer 1 showed a higher insertion AUC, and Layer 2, a higher overall faithfulness delta, with no statistically significant faithfulness difference among Layers 1, 2, and 4 (Appendix A.5). For meningioma, only the decoder-assisted attribution combined localization with a positive faithfulness delta (+0.309), whereas glioma was localized by most layers. These findings demonstrate that resolution collapse is a meaningful failure mode for deep-layer explainability in volumetric medical imaging, and that reconstruction-based decoding offers a practical, annotation-free path to recovering spatially reliable attribution at full input resolution.

## Figures and Tables

**Table 1:** Comparison of representative recent glioma/meningioma classification studies.

Study	Approach	Task	Performance	Explainability
[16]	2D CNN	Glioma/meningioma/pituitary	99% acc.	None
[17]	2D CNN ensemble	4-class	AUC 0.998 (MEN)	None
[18]	3D CNN	Glioma grading	—	None
[19]	3D ResNet	Glioma grading + healthy	—	None
[20]	3D CNN ensemble	IDH/MGMT in glioma	AUC 0.94/0.77	IG + Shapley (qualitative)
[21]	2D CNN	Tumor detection	—	Grad-CAM + SHAP, expert review
[22]	3D CNN	7-class incl. MEN	93.35% acc.	Grad-CAM (qualitative)
This study	3D CNN (ResNet-34 + CBAM)	Glioma/meningioma	96% acc., AUC 0.993	Validated, decoder-assisted

**Table 2:** Explainability methods applied at each residual stage of the 3D ResNet-34 backbone, showing the progressive reduction in spatial resolution and the corresponding method used to address it.

Method	Resolution	Layer
Grad-CAM	32^3^	1
Grad-CAM	16^3^	2
Grad-CAM + SmoothGrad	8^3^	3
Decoder-assisted Grad-CAM	4^3^	4

**Table 3:** Four-fold cross-validation summary table.

Fold	Val Accuracy (%)	Val AUC
1	98.00	0.983
2	98.89	0.998
3	98.89	0.995
4	97.56	0.992
Mean	98.33	0.992
Std	±0.58	±0.005

**Table 4:** Classification report on the test set (positive class: glioma).

Precision	Recall (Sensitivity)	Specificity	F1-Score	Accuracy	Support
0.99	0.94	0.985	0.96	0.96	450

**Figure A1:**
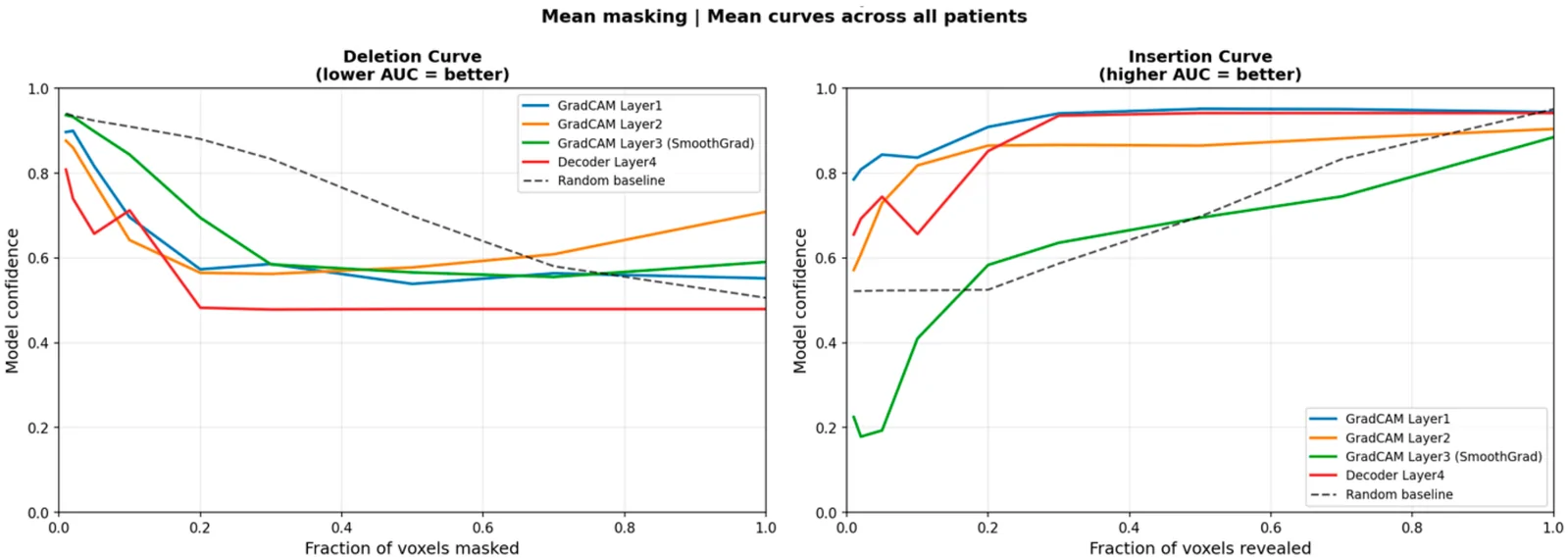
Overall insertion/deletion curves (n = 450).

**Figure A2:**
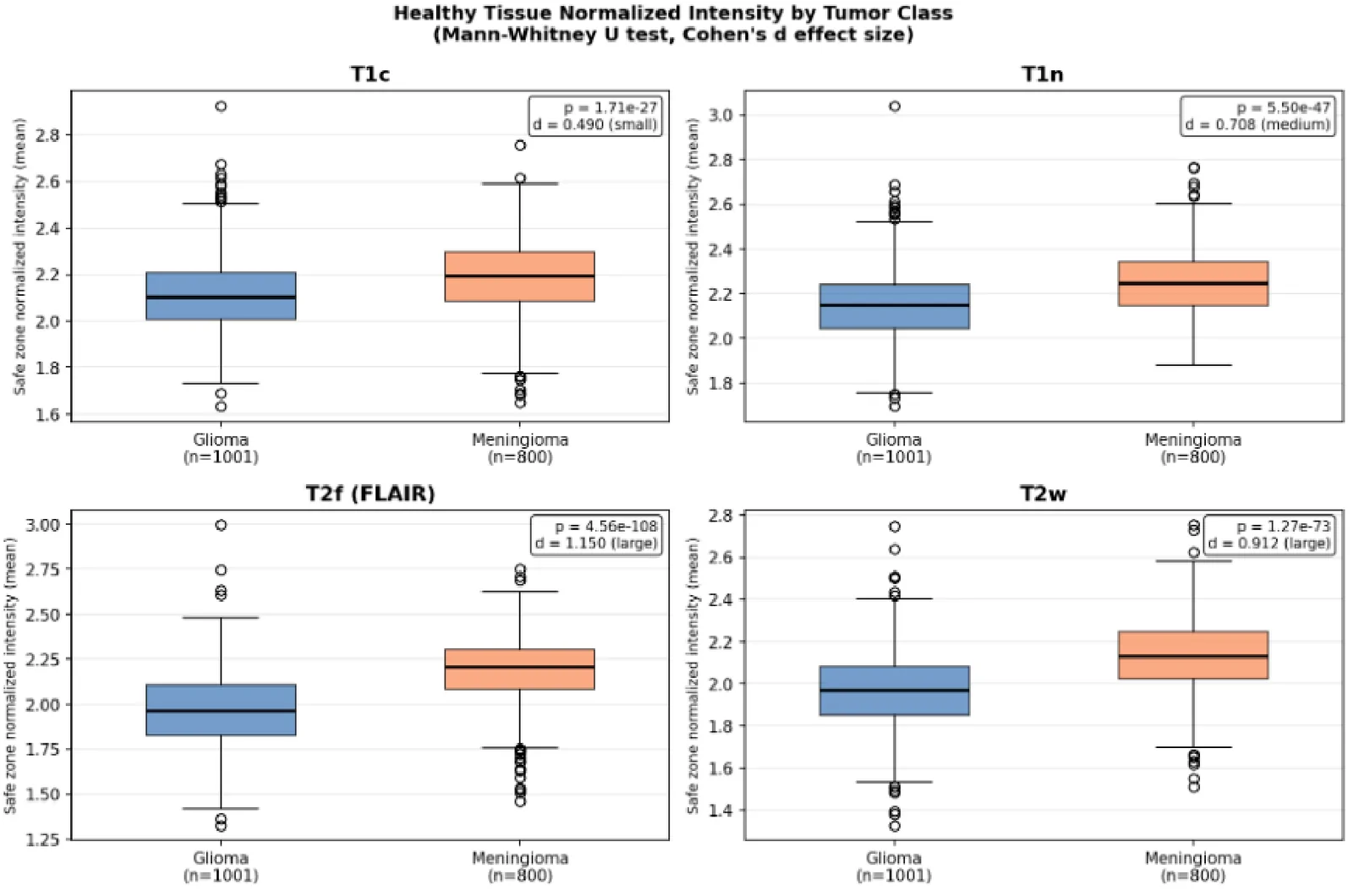
Box-plot figure of the training/validation set annotated with *p*-values and Cohen’s d per panel.

**Figure A3:**
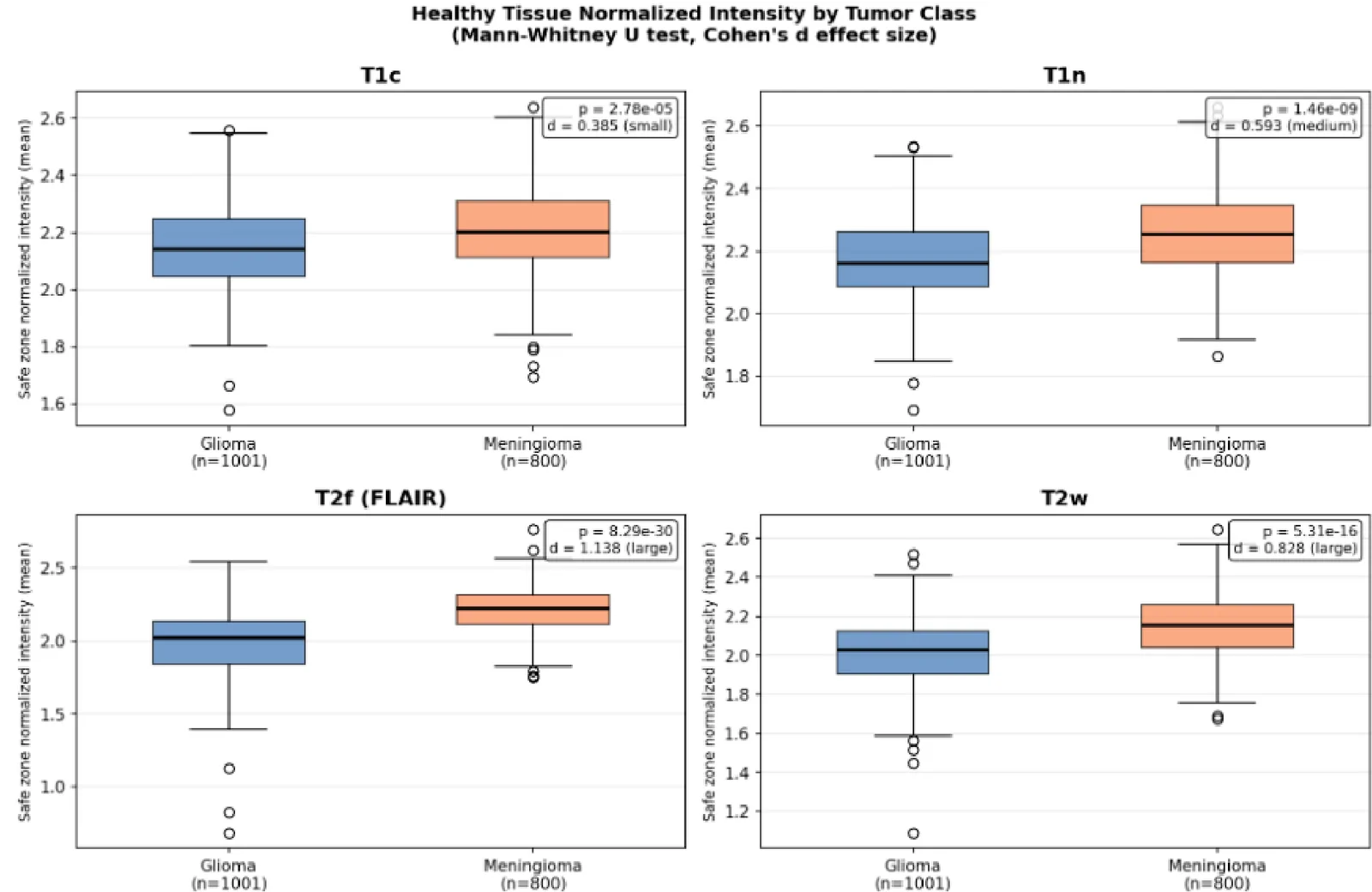
Box-plot figure of the test set annotated with *p*-values and Cohen’s d per panel.

## Data Availability

The original data presented in the study are openly available in [37,38,39].

## Abbreviations

The following abbreviations are used in this manuscript:

MRI	Magnetic Resonance Imaging
CNN(s)	Convolutional Neural Network(s)
AUC	Area Under the Curve
ROC	Receiver Operating Characteristic
CBAM	Convolutional Block Attention Module
ECE	Expected Calibration Error
T1c	T1-contrast enhanced
T1n	T1 native
T2f	T2-FLAIR
T2w	T2-weighted
XAI	Explainable Artificial Intelligence

## Appendix A

### Appendix A.1. Faithfulness/Perturbation Test

Full per-class results for the faithfulness/perturbation test summarized in Section 3.2.2 are provided in Table A1 and Table A2.

**Table A1:** Full per-class faithfulness/perturbation test results.

Method	Overall Δ	Faithful%	Glioma Δ	Glioma%	Meningioma Δ	Meningioma%
Layer 1	0.233	78.6%	0.440	98.2%	−0.011	55.4%
Layer 2	0.291	64.8%	0.548	100.0%	−0.010	23.3%
Layer 3	0.072	79.3%	0.117	100.0%	0.02	54.9%
Layer 4	0.217	91.2%	0.139	86.3%	0.309	96.9%

**Table A2:** Supplementary analyses on the best-prediction subset (n = 420), the complete test set (n = 450), and the remaining cases (n = 30) for faithfulness/perturbation test.

	n = 420	n = 450	Remaining (n = 30)
Layer 1	+0.233	+0.214	−0.052
Layer 2	+0.291	+0.269	−0.039
Layer 3	+0.072	+0.067	−0.003
Layer 4	+0.217	+0.198	−0.068

### Appendix A.2. Pointing Game

Full per-class pointing-game results summarized in Section 3.2.2 are provided in Table A3 and Table A4.

**Table A3:** Full per-class pointing-game results.

Method	Overall Acc	Ratio Above Random	Glioma	Meningioma
Layer 1	21.9%	~12×	31.3%	10.9%
Layer 2	6.4%	~3.5×	5.7%	7.3%
Layer 3	13.1%	~7×	20.7%	4.1%
Layer 4	80.2%	~45×	91.2%	67.4%

**Table A4:** Supplementary analyses on the best-prediction subset (n = 420), the complete test set (n = 450), and the remaining cases (n = 30) for pointing-game accuracy test.

	n = 420	n = 450	Remaining (n = 30)
Layer 1	21.9%	21.3%	13.3%
Layer 2	6.4%	7.3%	20.0%
Layer 3	13.1%	12.7%	6.7%
Layer 4	80.2%	80.2%	80.0%

### Appendix A.3. Insertion/Deletion Curves

Full overall and per-class insertion/deletion AUC results are provided in Table A5 and Table A6, with corresponding curves shown in Figure A1.

**Table A5:** Overall insertion/deletion AUC results (all 450 patients).

Method	Deletion AUC	Random	Insertion AUC	Random
Layer 1	0.584	0.702	0.919	0.702
Layer 2	0.616	0.703	0.851	0.703
Layer 3	0.62	0.705	0.655	0.703
Layer 4	0.506	0.703	0.887	0.704

**Table A6:** Per-class insertion/deletion AUC results (all 450 test patients, 250 glioma, and 200 meningioma).

Method	Glioma Del	Glioma Ins	Meningioma Del	Meningioma Ins
Layer 1	0.370	0.897	0.852	0.945
Layer 2	0.371	0.873	0.922	0.824
Layer 3	0.474	0.801	0.803	0.472
Layer 4	0.236	0.855	0.845	0.926

### Appendix A.4. Spearman Rank Correlation

Full pairwise Spearman rank correlation results are provided in Table A7 and Table A8.

**Table A7:** Pairwise Spearman rank correlation (n = 420 best predictions).

Pair	Overall ρ	Glioma ρ	Meningioma ρ
Layer 1 vs. Layer 2	0.616	0.639	0.589
Layer 1 vs. Layer 3	0.384	0.708	0.003
Layer 1 vs. Decoder	0.846	0.849	0.842
Layer 2 vs. Layer 3	0.364	0.606	0.080
Layer 2 vs. Decoder	0.565	0.607	0.515
Layer 3 vs. Decoder	0.357	0.718	−0.067

**Table A8:** Supplementary analyses on the best-prediction subset (n = 420), the complete test set (n = 450), and the remaining cases (n = 30) for spearman correlation ρ values.

	n = 420	n = 450	Remaining (n = 30)
Layer 1 vs. Layer 2	0.616	0.611	0.534
Layer 1 vs. Layer 3	0.384	0.383	0.362
Layer 1 vs. Decoder	0.846	0.796	0.104
Layer 2 vs. Layer 3	0.364	0.374	0.505
Layer 2 vs. Decoder	0.565	0.535	0.112
Layer 3 vs. Decoder	0.357	0.344	0.157

### Appendix A.5. Confidence Intervals and Statistical Significance

To determine whether the differences in attribution quality reported in Section 3.2 reflect robust trends rather than point estimates, we computed 95% confidence intervals via bootstrap resampling (1000 iterations) for each of the four validation metrics (faithfulness delta, pointing-game hit rate, deletion AUC, and insertion AUC) and each attribution layer. Pairwise comparisons between layers were tested using the Wilcoxon signed-rank test for continuous metrics and McNemar’s test for the binary pointing-game outcome, with the Holm–Bonferroni correction applied across all pairwise comparisons within each metric. Results are summarized in Table A9.

The decoder-assisted Grad-CAM at Layer 4 significantly outperformed all other layers on both the pointing-game hit rate and the deletion AUC (all *p* < 0.0001, Holm-corrected). On the insertion AUC, Layer 4 significantly outperformed Layers 1 and 3, but it was not distinguishable from Layer 2 (*p* = 0.479). On the faithfulness delta, Layer 3 was significantly lower than all other layers, while Layers 1, 2, and 4 were not significantly different from one another at the aggregate level; as detailed in Appendix A.6, Layer 4′s faithfulness advantage is concentrated in the meningioma subgroup rather than uniformly distributed across both classes.

**Table A9:** Mean value and 95% bootstrap confidence interval for each validation metric, by attribution layer. Faithfulness and pointing-game values are for the best-prediction subset (n = 420), while insertion and deletion AUCs are for the complete test set (n = 450).

Metric	Layer 1	Layer 2	Layer 3	Layer 4
Faithfulness delta	0.233 [0.199, 0.268]	0.291 [0.253, 0.331]	0.072 [0.054, 0.093]	0.217 [0.183, 0.251]
Pointing-game hit rate	0.219 [0.179, 0.260]	0.064 [0.041, 0.088]	0.131 [0.100, 0.162]	0.802 [0.762, 0.838]
Deletion AUC	0.584 [0.555, 0.613]	0.616 [0.586, 0.649]	0.620 [0.598, 0.642]	0.506 [0.476, 0.537]
Insertion AUC	0.919 [0.913, 0.924]	0.851 [0.836, 0.869]	0.655 [0.633, 0.678]	0.887 [0.879, 0.895]

### Appendix A.6. Boundary-Inclusive Pointing Game

The standard pointing-game metric counts a hit only when the peak attribution voxel falls strictly within the tumor segmentation mask. Since meningiomas are extra-axial lesions for which relevant diagnostic context (dural attachment and adjacent brain displacement) can plausibly extend beyond the tumor boundary, we tested whether this strict criterion disproportionately penalizes meningioma attribution. Tumor masks were dilated isotropically by 2, 3, 5, and 10 mm using a 26-connected structuring element, and the pointing-game hit rate was recomputed against each dilated mask. Results are shown in Table A10.

Layer 4’s meningioma hit rate remained essentially flat across all tested margins (67.4% strict to 68.9% at +10 mm), indicating that the strict criterion is not an artificially narrow or unfair standard for this layer: where its attribution misses the tumor for meningioma cases, it does so by a substantial margin rather than by a small boundary offset. Layers 1–3 showed comparatively larger gains at wider margins, consistent with their broader and less spatially precise attribution patterns.

**Table A10:** Pointing-game hit rate under progressive mask dilation (strict, +2 mm, +3 mm, +5 mm, and +10 mm). (Best prediction subset, n = 420).

Layer	Strict	+2 mm	+3 mm	+5 mm	+10 mm
Layer 4—Overall	80.2%	80.5%	80.5%	80.5%	81.0%
Layer 4—Glioma	91.2%	91.2%	91.2%	91.2%	91.2%
Layer 4—Meningioma	67.4%	67.9%	67.9%	67.9%	68.9%
Layer 3—Glioma	20.7%	30.4%	35.2%	42.3%	55.1%
Layer 3—Meningioma	4.1%	6.2%	8.8%	9.3%	15.0%

### Appendix A.7. Ablation of Decoder Inputs

**Table A11:** Pointing-game hit rate under Layer 4/skip-connection ablation. (Best prediction subset, n = 420).

Condition	Overall	Glioma	Meningioma
Baseline	80.2%	91.2%	67.4%
Layer 4 zeroed	71.7%	79.7%	62.2%
Layer 4 mean-filled	71.7%	79.7%	62.2%
All-skip connections zeroed	0.0%	0.0%	0.0%
All-skip connections mean-filled	0.0%	0.0%	0.0%
Skip1 only zeroed	8.6%	9.3%	7.8%
Skip2 only zeroed	73.1%	78.4%	66.8%
Skip3 only zeroed	71.9%	79.7%	62.7%

**Table A12:** Faithfulness delta under Layer 4/skip-connection ablation. (Best prediction subset, n = 420).

Condition	Overall	Glioma	Meningioma
Baseline	0.217	0.139	0.309
Layer 4 zeroed	0.396	0.006	0.856
Layer 4 mean-filled	0.396	0.006	0.856
All skip connections zeroed	−0.013	0.004	−0.033
All skip connections mean-filled	−0.014	0.005	−0.035
Skip1 only zeroed	0.288	−0.001	0.628
Skip1 only mean-filled	0.157	−0.001	0.343

### Appendix A.8. Adversarial Robustness

**Table A13:** Classifier flip rate and attribution stability under FGSM perturbation, with random-noise control. Flip rates are computed on the best-prediction subset (n = 420). The n for the attribution Spearman correlation is the number of unflipped patients shown in the column.

Ε	Flip Rate—Overall	Flip Rate—Glioma	Flip Rate—Meningioma	Mean Attribution Spearman (Unflipped)	Random-Noise Control Flip Rate
0.01	6.9%	5.7%	8.3%	0.757 (n = 391)	0.0%
0.03	56.7%	33.9%	83.4%	0.757 (n = 182; menin. n = 32)	0.0%
0.05	74.3%	53.7%	98.5%	0.759 (n = 108; menin. n = 3)	0.0%

## Appendix B. Healthy Tissue Intensity Statistics by Cohort

### Appendix B.1. Procedure

For each of the 1801 training/validation and 450 test patients, a brain mask was defined as the union of non-zero voxels across the four modalities. The tumor segmentation (BraTS 2023) was binarized and dilated by 20 voxels (approximately 20 mm), and the healthy-tissue region (“safe zone”) was defined as brain voxels outside the dilated mask. Volumes were resized to 128 × 128 × 128, and z-scores were normalized, exactly as in training. The safe-zone mask was resized by nearest-neighbor interpolation. For each patient and modality, the mean normalized intensity within the safe zone was recorded. The glioma and meningioma cohorts were compared with the Mann–Whitney U test, effect sizes are reported as Cohen’s d, and the comparison was repeated after randomly permuting the class labels as a sanity check on the procedure.

### Appendix B.2. Results

Cohen’s d values for the training/validation and held-out test cohorts are given in Table A14; *p*-values for the training/validation comparison ranged from 1.7 × 10^−27^ (T1c) to 4.6 × 10^−108^ (T2f). With randomly permuted class labels, no modality showed an association (*p* > 0.49 in the training/validation data and *p* > 0.35 in the test data).

**Table A14:** Mean normalized safe-zone intensity (SD) and effect sizes by modality.

Modality	Glioma Train/Val	Meningioma Train/Val	Cohen’s d Train/Val	Cohen’s d Test	*p* Train/Val
T1c	2.109 (0.152)	2.186 (0.162)	0.490	0.385	1.7 × 10^−27^
T1n	2.145 (0.147)	2.247 (0.141)	0.708	0.593	5.5 × 10^−47^
T2f	1.962 (0.205)	2.186 (0.182)	1.150	1.138	4.6 × 10^−108^
T2w	1.969 (0.180)	2.129 (0.171)	0.912	0.828	1.3 × 10^−73^

*Normalization-parameter probe*.

### Appendix B.3. Procedure

For each of the 1801 training/validation and 450 test patients, the mean and standard deviation of each of the four modalities were computed on the 128 × 128 × 128 resized volume before z-scoring, exactly as in the training pipeline (eight parameters). A logistic regression (standardized features and balanced class weights) was cross-validated 5-fold on the training/validation patients and evaluated once on the test set, with a bootstrap 95% confidence interval and a label-shuffle control (mean over 20 permutations). For comparison, models were fitted on lesion volume (tumor plus oedema, from the BraTS segmentation), brain volume, both volumes, the parameters plus both volumes, and the parameters after regressing out both volumes.

### Appendix B.4. Results

With the eight normalization parameters as the only inputs, the AUC on the held-out test set was close to 0.5 (0.506; 95% CI, 0.451 to 0.560; label-shuffled control, 0.480); in cross-validation on the training/validation patients, it was 0.600 (Table A15). Lesion volume alone gave a test AUC of 0.814 (95% CI, 0.772 to 0.855). The single-parameter AUCs lay between 0.43 and 0.49 in the training/validation data and between 0.51 and 0.58 in the test set. After regressing out lesion and brain volume, the parameters gave a cross-validated AUC of 0.678 and a test AUC of 0.594 (95% CI, 0.541 to 0.649).

**Table A15:** Prediction of tumor class from normalization parameters, and lesion and brain volume (logistic regression; AUC, training/validation 5-fold cross-validation and held-out test set with bootstrap 95% CI).

Feature Set	Cross-Validated AUC, Training/Validation	Test AUC (95% CI)
Mean and SD, 4 modalities (8 parameters)	0.600	0.506 (0.451 to 0.560)
Lesion volume only	0.787	0.814 (0.772 to 0.855)
Brain volume only	0.673	0.639 (0.587 to 0.687)
Lesion and brain volume	0.806	0.823 (0.781 to 0.864)
Parameters and both volumes	0.828	0.830 (0.790 to 0.872)
Parameters with both volumes regressed out	0.678	0.594 (0.541 to 0.649)

## References

[1] Ortega M.A., Fraile-Martinez O., Boaru D.L. (2026). From Classical to Emerging Biomarkers of Brain and Central Nervous System Tumors. An Evidence-Based Review with a Focus on Gliomas. Cell. Mol. Neurobiol..

[2] Ostrom Q.T., Francis S.S., Barnholtz-Sloan J.S. (2021). Epidemiology of brain and other CNS tumors. Curr. Neurol. Neurosci. Rep..

[3] Juncà A., Escrichs A., Martín I. (2026). Impact of meningioma and glioma on whole-brain dynamics. Sci. Rep..

[4] Zong H., Parada L.F., Baker S.J. (2015). Cell of origin for malignant gliomas and its implication in therapeutic development. Cold Spring Harb. Perspect. Biol..

[5] Song D., Cai R., Lou Y. (2025). Advancements in the application of MRI radiomics in meningioma. Radiat. Oncol..

[6] Gorenshtein A., Liba T., Goren A. (2025). Lightweight transfer learning models for Multi-Class brain tumor classification: Glioma, meningioma, pituitary tumors, and no tumor MRI screening. J. Imaging Inform. Med..

[7] Taha A.M., Aly S.A., Darwish M.F. (2025). Detecting Glioma, Meningioma, and Pituitary Tumors, and Normal Brain Tissues based on Yolov11 and Yolov8 Deep Learning Models. arXiv.

[8] Zhang J., Zhang G., Cao Y., Ren J., Zhao Z., Han T., Chen K., Zhou J. (2022). A Magnetic Resonance Imaging-Based Radiomic model for the noninvasive preoperative differentiation between transitional and atypical meningiomas. Front. Oncol..

[9] Ahmad B., Sun J., You Q. (2022). Brain tumor classification using a combination of variational autoencoders and generative adversarial networks. Biomedicines.

[10] Louis D.N., Perry A., Reifenberger G. (2016). The 2016 World Health Organization Classification of Tumors of the Central Nervous System: A summary. Acta Neuropathol..

[11] Zikou A.K., Romeo E., Alexiou G.A. (2026). The role of advanced MR imaging in Gliomas. Appl. Sci..

[12] Xu N., Ma W., Dong W. (2026). A scoping review of artificial intelligence applications in meningioma from image analysis to prognostic prediction. Discov. Oncol..

[13] Kamepalli H., Kalaparti V., Kesavadas C. (2023). Imaging recommendations for the diagnosis, staging, and management of adult brain tumors. Indian J. Med. Paediatr. Oncol..

[14] Singh A., Sengupta S., Lakshminarayanan V. (2020). Explainable deep learning models in medical image analysis. J. Imaging.

[15] Peng C., Li L., Peng D. (2025). A Method-Oriented Review of Explainable Artificial intelligence for Neurological medical Imaging. Expert Syst..

[16] Al-Zoghby A.M., Al-Awadly E.M.K., Moawad A., Yehia N., Ebada A.I. (2023). Dual Deep CNN for Tumor Brain Classification. Diagnostics.

[17] Liu K.-Y., Lu N.-H., Huang Y.-H., Matsushima A., Kimura K., Okamoto T., Chen T.-B. (2025). Majority Voting Ensemble of Deep CNNs for Robust MRI-Based Brain Tumor Classification. Diagnostics.

[18] Mzoughi H., Njeh I., Wali A., Ben Slima M., BenHamida A., Mhiri C., Ben Mahfoudhe K. (2020). Deep Multi-Scale 3D Convolutional Neural Network (CNN) for MRI Gliomas Brain Tumor Classification. J. Digit. Imaging.

[19] Chatterjee S., Nizamani F.A., Nürnberger A., Speck O. (2022). Classification of Brain Tumours in MR Images Using Deep Spatiospatial Models. Sci. Rep..

[20] Christodoulou R.C., Vamvouras G., Pitsillos R., Roka K., Solomou E.E., Kalogeropoulou C., Zampakis P., Georgiou M.F. (2026). Externally Validated Explainable 3D CNN Ensemble Model for Non-Invasive Prediction of IDH and MGMT Status in Gliomas. Front. Oncol..

[21] Gharaibeh N. (2025). Enhancing Interpretability in Brain Tumor Detection: Leveraging Grad-CAM and SHAP for Explainable AI in MRI-Based Cancer Diagnosis. Appl. Comput. Sci..

[22] Chakrabarty S., Sotiras A., Milchenko M., LaMontagne P., Hileman M., Marcus D. (2021). MRI-Based Identification and Classification of Major Intracranial Tumor Types by Using a 3D Convolutional Neural Network: A Retrospective Multi-Institutional Analysis. Radiol. Artif. Intell..

[23] Brima Y., Atemkeng M. (2024). Saliency-driven explainable deep learning in medical imaging: Bridging visual explainability and statistical quantitative analysis. BioData Min..

[24] Chinga A., Bendezu W., Angulo A. (2025). Comparative Study of CNN Architectures for Brain Tumor Classification using MRI: Exploring GradCAM for visualizing CNN Focus. Eng. Proc..

[25] Wall C., Wang L., Rizk R. (2025). Winsor-CAM: Human-Tunable Visual Explanations from Deep Networks via Layer-Wise Winsorization. arXiv.

[26] Singh A.K., Chaudhuri D., Singh M.P. (2024). Integrative CAM: Adaptive layer fusion for comprehensive interpretation of CNNs. arXiv.

[27] Gao Y., Liu J., Li W. (2023). Augmented Grad-CAM++: Super-Resolution saliency Maps for visual interpretation of deep neural network. Electronics.

[28] Santos R., Pedrosa J., Mendonça A.M., Campilho A. (2025). Grad-CAM: The impact of large receptive fields and other caveats. Comput. Vis. Image Underst..

[29] Guo X., Hou B., Yang C., Ma S., Ren B., Wang S., Jiao L. (2023). Visual explanations with detailed spatial information for remote sensing image classification via channel saliency. Int. J. Appl. Earth Obs. Geoinf..

[30] Dudeja T., Sharma P. (2026). Geometry-aware localization evaluation of grad-CAM for wafer map defect classification. Sci. Rep..

[31] Vinogradova K., Dibrov A., Myers G. Towards interpretable semantic segmentation via Gradient-Weighted Class Activation Mapping (Student Abstract). Proceedings of the AAAI Conference on Artificial Intelligence.

[32] Fu S., Hamilton M., Brandt L., Feldman A., Zhang Z., Freeman W.T. (2024). FeatUp: A Model-Agnostic framework for features at any resolution. arXiv.

[33] Uchiyama T., Sogi N., Niinuma K., Fukui K. Visually explaining 3D-CNN predictions for video classification with an adaptive occlusion sensitivity analysis. Proceedings of the IEEE/CVF Winter Conference on Applications of Computer Vision (WACV).

[34] Kibriya H., Amin R., Kim J., Nawaz M., Gantassi R. (2023). A novel approach for brain tumor classification using an ensemble of deep and Hand-Crafted features. Sensors.

[35] Liu Y., Shin S., Lee H. (2026). NeURoSPFNET: Vision–Language Semantic Prior-Guided Frequency-Enhanced Network for Brain Tumor Segmentation in Multimodal MRI. Sensors.

[36] Thenuwara G., Curtin J., Tian F. (2023). Advances in diagnostic tools and therapeutic Approaches for Gliomas: A Comprehensive review. Sensors.

[37] Baid U., Ghodasara S., Mohan S., Bilello M., Calabrese E., Colak E., Farahani K., Kalpathy-Cramer J., Kitamura F.C., Pati S. (2021). The RSNA-ASNR-MICCAI BRATS 2021 Benchmark on Brain Tumor Segmentation and Radiogenomic Classification. arXiv.

[38] LaBella D., Adewole M., Alonso-Basanta M. (2023). The ASNR-MICCAI Brain Tumor Segmentation (BRATS) Challenge 2023: Intracranial Meningioma. arXiv.

[39] Bakas S., Akbari H., Sotiras A., Bilello M., Rozycki M., Kirby J.S., Freymann J.B., Farahani K., Davatzikos C. (2017). Advancing the Cancer Genome Atlas glioma MRI collections with expert segmentation labels and radiomic features. Sci. Data.

[40] Rohlfing T., Zahr N.M., Sullivan E.V., Pfefferbaum A. (2009). The SRI24 multichannel atlas of normal adult human brain structure. Hum. Brain Mapp..

[41] Chen S., Ma K., Zheng Y. (2019). Med3D: Transfer Learning for 3D medical Image analysis. arXiv.

[42] He K., Zhang X., Ren S., Sun J. Deep Residual Learning for Image Recognition. Proceedings of the IEEE Conference on Computer Vision and Pattern Recognition (CVPR).

[43] Woo S., Park J., Lee J.-Y., Kweon I.S. (2018). CBAM: Convolutional Block Attention Module. Computer Vision—ECCV 2018.

[44] Szegedy C., Vanhoucke V., Ioffe S., Shlens J., Wojna Z. Rethinking the Inception Architecture for Computer Vision. Proceedings of the 2016 IEEE Conference on Computer Vision and Pattern Recognition.

[45] Cui Y., Jia M., Lin T.-Y., Song Y., Belongie S. Class-Balanced Loss Based on Effective Number of Samples. Proceedings of the IEEE Computer Society Conference on Computer Vision and Pattern Recognition.

[46] Kingma D.P., Ba J. (2014). Adam: A method for stochastic optimization. arXiv.

[47] Pascanu R., Mikolov T., Bengio Y. (2012). On the difficulty of training Recurrent Neural Networks. arXiv.

[48] Selvaraju R.R., Cogswell M., Das A., Vedantam R., Parikh D., Batra D. (2016). Grad-CAM: Visual Explanations from Deep Networks via Gradient-based Localization. arXiv.

[49] Smilkov D., Thorat N., Kim B., Viégas F., Wattenberg M. (2017). SmoothGrad: Removing noise by adding noise. arXiv.

[50] Samek W., Binder A., Montavon G., Bach S., Müller K.-R. (2015). Evaluating the visualization of what a Deep Neural Network has learned. arXiv.

[51] Zhang J., Bargal S.A., Lin Z., Brandt J., Shen X., Sclaroff S. (2017). Top-Down neural attention by excitation backprop. Int. J. Comput. Vis..

[52] Petsiuk V., Das A., Saenko K. (2018). RISE: Randomized Input Sampling for explanation of black-box models. arXiv.

[53] Parodi S., Verda D., Bagnasco F., Muselli M. (2022). The clinical meaning of the area under a receiver operating characteristic curve for the evaluation of the performance of disease markers. Epidemiol. Health.

[54] Guo C., Pleiss G., Sun Y., Weinberger K.Q. (2017). On calibration of modern neural networks. arXiv.

[55] Bonneville F., Jäger H.R., Smirniotopoulos J.G. (2024). Differential diagnosis of intracranial masses. Diseases of the Brain, Head and Neck, Spine 2024–2027: Diagnostic Imaging.

[56] Jiang P.-T., Zhang C.-B., Hou Q., Cheng M.-M., Wei Y. (2021). LayerCAM: Exploring Hierarchical Class Activation maps for Localization. IEEE Trans. Image Process..

[57] Li K., Wu Z., Peng K.-C., Ernst J., Fu Y. (2018). Tell me where to look: Guided attention inference network. arXiv.

[58] Rapalino O., Smirniotopoulos J.G. (2016). Extra-axial brain tumors. Handbook of Clinical Neurology.

[59] Bouhafra S., Bahi H.E. (2024). Deep Learning Approaches for Brain Tumor Detection and Classification using MRI Images (2020 to 2024): A Systematic review. J. Imaging Inform. Med..

[60] Babar N.A., Lateef J., Syed S., Dietlmeier J., O’Connor N.E., Raupp G.B., Spanias A. (2025). Brain tumor classification in MRI scans using edge computing and a shallow Attention-Guided CNN. Biomedicines.

